# Transcriptional insights into the pyramided resistance to rice bacterial blight

**DOI:** 10.1038/s41598-018-29899-1

**Published:** 2018-08-17

**Authors:** Lifen Gao, Zhiwei Fang, Junfei Zhou, Lun Li, Long Lu, Lili Li, Tiantian Li, Lihong Chen, Weixiong Zhang, Wenxue Zhai, Hai Peng

**Affiliations:** 1 Institute for Systems Biology, Jianghan University, Wuhan, Hubei 430056 China; 2 Institute of Genetics and Developmental Biology, Chinese Academy of Sciences, Beijing, 100101 China

## Abstract

The pyramiding of resistance (R) genes provides broad-spectrum and durable resistance to plant diseases. However, the genetic basis for bacterial blight (BB) resistance remains unclear. The BB R gene pyramided line IRBB54, which expresses *xa5* and *Xa21*, possessed a higher level of resistance than both single *R* gene lines. Large-scale genotyping of genetic markers in this study revealed similar genetic backgrounds among the near-isogenic lines (NILs), suggesting that resistance in the resistant NILs was mainly conferred by the individual R genes or the interaction between them. Transcriptome analysis demonstrated that more than 50% of the differentially expressed genes (DEGs), and more than 70% of the differentially expressed functions, were shared between IRBB54 and IRBB5 or IRBB21. Most of the DEGs in the resistant NILs were downregulated and are predicted to function in cellular and biological process. The DEGs common among the resistant NILs mainly showed non-additive expression patterns and enrichment in stress-related pathways. The differential expression of agronomic trait-controlled genes in the resistant NILs, especially in IRBB54, indicated the existence of potential side-effects resulting from gene pyramiding. Our findings contribute to the understanding of R gene pyramiding, as well as its effects on targeted and non-targeted trait(s).

## Introduction

Rice (*Oryza sativa* L.) is the staple food of more than half of the world’s population. Bacterial blight (BB), caused by *Xanthomonas oryzae* pv. *oryzae* (*Xoo*), is one of the most serious rice diseases affecting major rice-growing regions globally. Host plant resistance is currently the most effective and economical way to control BB^1^. To date, approximately 40 BB-resistance (R) genes (*Xa* genes) have been identified in rice, 10 of which (*Xa1*^2^, *Xa3/Xa26*^3^, *Xa4*^4^, *xa5*^5,6^, *Xa10*^7^, *xa13*^8^, *Xa21*^9^, *Xa23*^10^, *xa25*^11^, and *Xa27*^12^) have been cloned. These R genes have been widely deployed in breeding programs, but some pathogen strains have overcome plant resistance conferred by a single R gene. For example, strains with mutated *avrXa4* overcame the resistance mediated by *Xa4*^T^^13^ and *xa5* is ineffective against strains that possess the transcription activator-like (TAL) effector gene *pthXo1*^14^.

The pyramiding of multiple genes into a single genotype is considered an effective way to develop offspring with elite performance in target traits. The typical application of gene pyramiding is to breed varieties with broad-spectrum and durable disease resistance; for example, the pyramided rice lines harboring the R genes *Xa4* + *Xa21*, *xa5* + *Xa21*, *Xa4* + *xa5* + *Xa21*, *xa5* + *xa13* + *Xa21*^15^, or *xa5* + *Xa4*^16^ have a wider spectrum or higher level of resistance than each R-gene line alone. Interestingly, the line with *xa5* and *Xa27* stacked had attenuated BB resistance as compared to the line harboring only *Xa27*^17^. Similarly, both *avrXa10*-dependent *Xa10* expression and *Xa10*-mediated resistance to PXO99^A^ were partially suppressed in *xa5* and *Xa10* double homozygous plants^7^; in addition, *AvrXa23-*induced *Xa23* expression was abolished, and the level of *Xa23*-mediated resistance weakened, in *xa5* homozygous plants^18^.

Gene pyramiding is a time-consuming process and hence understanding the underlying molecular mechanisms is necessary to avoid invalid or antagonistic gene combinations. For this, near-isogenic-lines (NILs) with pyramids of the R genes, developed by multi-generations backcrossing, are valuable tools for studying the effects of single R genes and R gene combinations^15,19^; high-throughput sequencing is also a powerful tool that can be used to analyze the transcriptional effects of gene pyramiding, providing expression information on a genome-wide scale. In this study, we profiled the transcriptomes of two lines, each with a single R gene, and a pyramided line with both R genes, to answer the following questions: Are the effects of R genes accumulative or synergistic in the pyramided line? Can the accumulative effect of the induced genes explain the broad and durable resistance that is widely observed in pyramided lines? Finally, does the pyramiding strategy promote side effects, in addition to the advantages related to BB resistance?

## Results

### Validation of the resistant NILs

**Figure 1:**
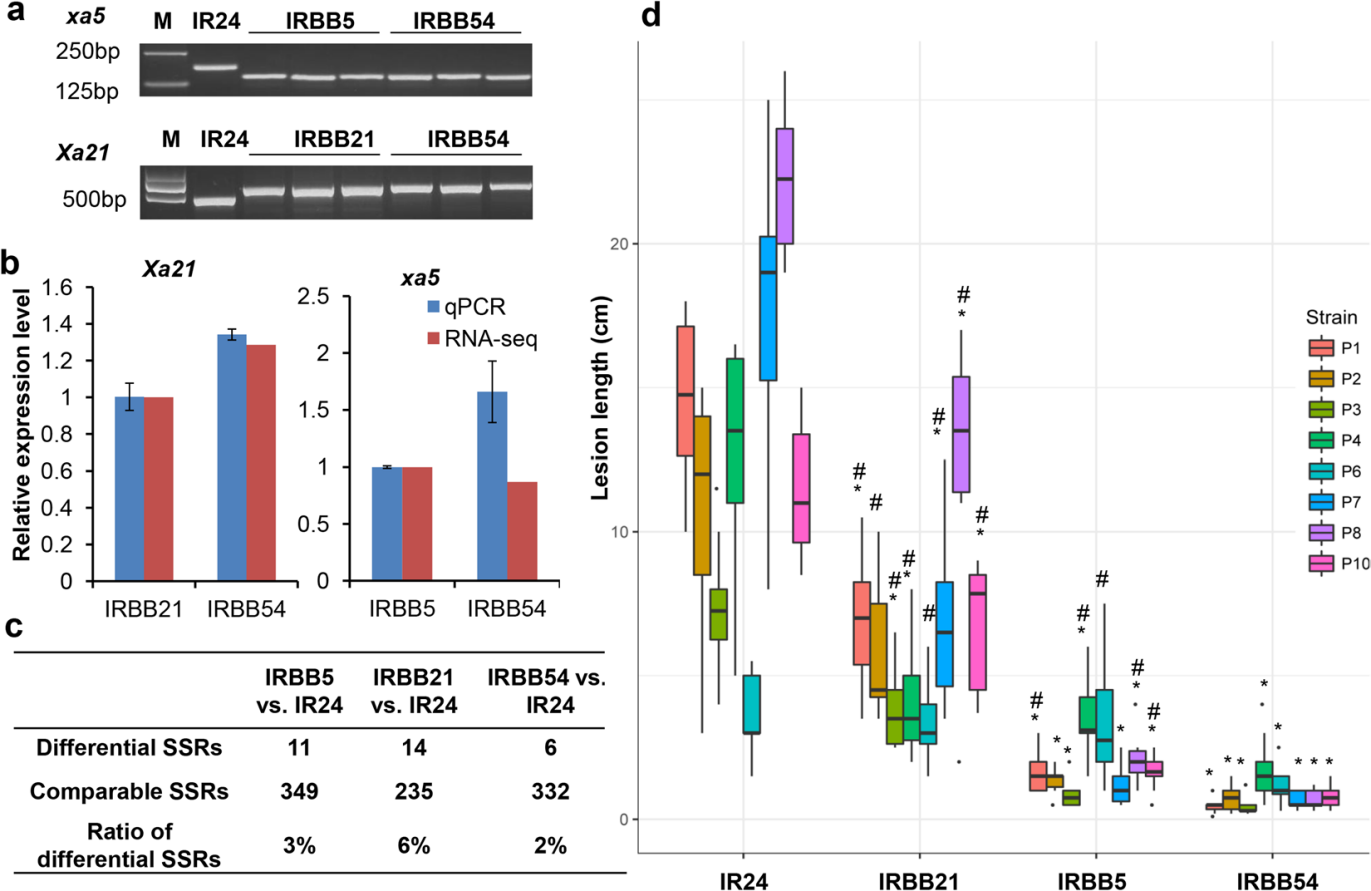
Validation of the three resistant NILs, IRBB5, IRBB21, and IRBB54. (**a**) Validation of *Xa21* and *xa5* in the genomes of the resistant NILs using molecular markers. Full-length gels are presented in Supplemental Fig. 4. (**b**) The relative expression level of *Xa21* and *xa5* in the resistant NILs identified by RNA-seq and qPCR. IR24 was the reference sample and rice *ubiquitin* gene was the internal control. (**c**) Genetic background analysis of the three resistant NILs by SSR markers. (**d**) Lesion lengths of the three resistant NILs after inoculation with eight *Xoo* strains. P1–P10: *Xoo* strains. ^·^Represent the outliers. *Represents a significance level of *p* < 0.01 in IRBB5, IRBB21, and IRBB54 with respect to IR24 (*Student’s t test*, two-tailed). ^#^Represents a significance level of *p* < 0.01 in IRBB5 and IRBB21 with respect to IRBB54 (*Student’s t test*, two-tailed).

Genetic background can influence BB resistance, as was shown in the pyramiding line with *xa5* + *xa13* + *xa21*^20^. Through backcrossing, the R genes *xa5*, *xa21*, and *xa5* + *xa21* were introduced into the same genetic background of IR24 to develop the BB resistant NILs of IRBB5, IRBB21 and IRBB54. The introduction of R genes into the three resistant NILs was validated through linked molecular markers (Fig. 1a) and the relative expression of R genes in the resistant NILs, with respect to the recipient line IR24 was measured by qRT-PCR using the *ubiquitin* gene as an internal control^21^ (Fig. 1b). As many as 3,105 simple sequence repeats (SSRs), including forty-eight SSRs listed in the National Agricultural Standard of China (NY/T 1433–3014) and 3,057 randomly-selected SSRs from the *Japonica* reference genome (irgsp1.0), were chosen as target SSRs to evaluate the similarity in the genetic background of the three resistant NILs. The Ampseq-SSR genotyping method, developed in our previous study^22^, was used to genotype the 3105 SSRs in the NILs. Through strict quality control of the raw reads (see methods), we found that the qualified reads for each sample were low, leading to a low coverage of the 3105 target SSRs. However, we then searched for comparable SSRs for the three groups of samples (IRBB5 vs. IR24, IRBB21 vs. IR24, and IRBB54 vs. IR24) following the criterions for valid SSR and comparable SSR (see methods), and identified 349, 235, and 332 comparable SSRs in sample groups IRBB5 vs. IR24, IRBB21 vs. IR24, and IRBB54 vs. IR24, respectively, which were more than the 48 SSRs adopted by the National Agricultural Standard of China to identify rice varieties (NY/T 1433–3014) (Fig. 1c). The genotypes of over 94% of the comparable SSR markers in the resistant NILs were identical to the IR24 donor (Fig. 1c, Supplementary Table S1), suggesting the similarity of the genetic backgrounds between the resistant NILs and IR24. The region linked to *xa5* covered approximately seven megabase pairs in IRBB5 and IRBB54, as revealed by the peak of single nucleotide polymorphism (SNP) distribution of the expressed genes identified from the transcriptome profiles of the resistant NILs (Supplementary Fig. S1). The SNPs outside the R gene-linked regions were sparsely distributed, reinforcing the conclusion that the genetic backgrounds were similar among the three resistant NILs. Therefore, the three resistant NILs and the susceptible line IR24 formed an optimal system to compare the resistance mechanisms from the single R genes and R gene combinations.

### Broader and stronger resistance of the pyramided line

The ten commonly used *Xoo* strains from the Philippines were selected for resistance evaluation. Because the morphology of the P5 and P9 strains was abnormal, only the remaining eight strains were used. Among them, P1, P2, and P6 have traditionally been used for the functional identification of *Xa4*^23^, *xa5*^5^, and *Xa21*^9^, respectively. IR24 was susceptible to all eight tested *Xoo* strains and developed the longest lesions with the P8 strain (Fig. 1d, Supplementary Fig. S2), indicating that these tested strains were virulent and effective for resistance testing. IRBB5 was resistant to six strains, but only moderately resistant to strains P4 and P6 (Fig. 1d, Supplementary Fig. S2) which harbor the *pthXo1* effector^24^ and therefore, partially overcame the resistance conveyed by *xa5*^14^.

*Xa21* was the first BB resistance gene to be cloned and has been found to confer resistance to a wide spectrum of *Xoo* strains^9^. In the present study, however, IRBB21 exhibited moderate resistance to P2, P3, P4, and P6; moderate susceptibility to P1, P7, and P10; and full susceptibility to the P8 strain, with a mean lesion length of approximately 13 cm (Fig. 1d, Supplementary Fig. S2). Compared to the two lines with a single R gene, the pyramided line IRBB54 showed a higher level and wider spectrum of resistance to the eight *Xoo* strains (Fig. 1d, Supplementary Fig. S2), which is consistent with a previous report^15^.

### DEG pyramiding and BB resistance

As shown by the qRT-PCR analysis (Fig. 1b) and previous reports^6,25^, both *xa5* and *Xa21* were constitutively expressed, suggesting that their resistance mechanisms could be detected before *Xoo* infection. In the present study, the transcriptomes of IRBB5, IRBB21, IRBB54, and the susceptible line IR24 were analyzed by RNA-seq, before *Xoo* infection. Through quality control for all raw reads (see methods), the clean reads that mapped to the reference genome for each sample ranged from 2,472,094 to 4,783,943. A total of 55,779 genes were covered. Only 2.3–9.6% of the total mapped reads were multiple alignments. This indicated that a reliable result could be achieved based on these data.

**Figure 2:**
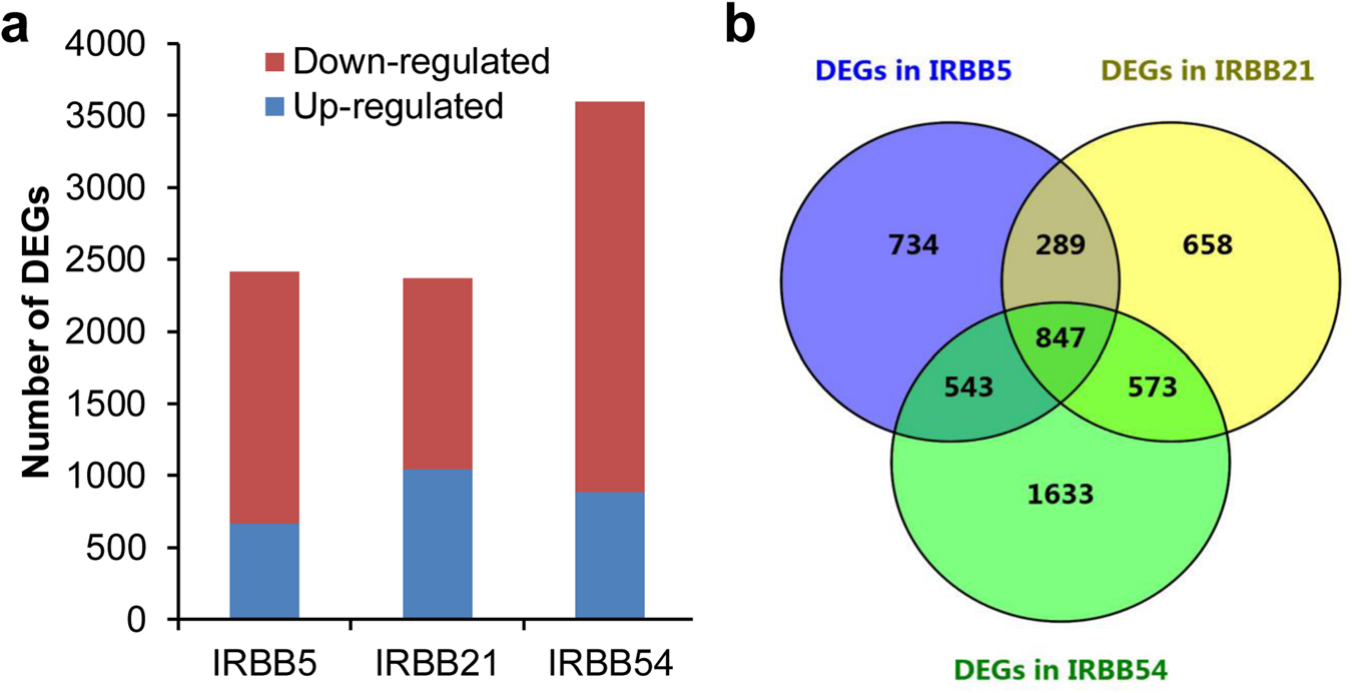
DEGs in the three resistant NILs. **(a**) Numbers of up- and downregulated DEGs. (**b**) Venn diagram of DEGs.

The RNA-seq data also indicated that *Xa21* and *xa5* were constitutively expressed (Fig. 1b). Compared to the susceptible line IR24, 2,367, 2,412, and 3,596 DEGs were identified from IRBB21, IRBB5, and IRBB54, respectively, before *Xoo* infection (Fig. 2a, Dataset 1). These data imply that the R genes cause broad disturbances at the transcriptional level. The data also showed that the R genes tended to suppress rather than upregulate the expression of their downstream genes (Fig. 2a). *Xa21* and *xa5* regulated almost the same number of DEGs (2,367 and 2,412, respectively), of which 1,136 (~50%) were common between them, indicating the functional redundancy and independence of the two R genes (Fig. 2b).

The pyramiding of *Xa21* and *xa5* induced substantially more DEGs than either of the two R genes alone (Fig. 2a), providing a larger transcriptional pool with which to resist various *Xoo* strains. Interestingly, 1,420 (60%) and 1,390 (58%) of the DEGs induced by *xa5* and *Xa21*, respectively, were also induced in the pyramided line, including 543 *xa5*-specific and 573 *Xa21*-specific DEGs (Fig. 2b). These observations indicated that the transcriptional mechanisms adopted by the single R genes were partially combined in the pyramided line. Except for the DEGs common among the NILs, 1,633 (45%) of the 3,596 DEGs were specific to the pyramided line (Fig. 2b), suggesting that new BB resistance mechanisms were acquired through interactions between *xa5* and *Xa21*.

### DEF pyramiding and BB resistance

**Figure 3:**
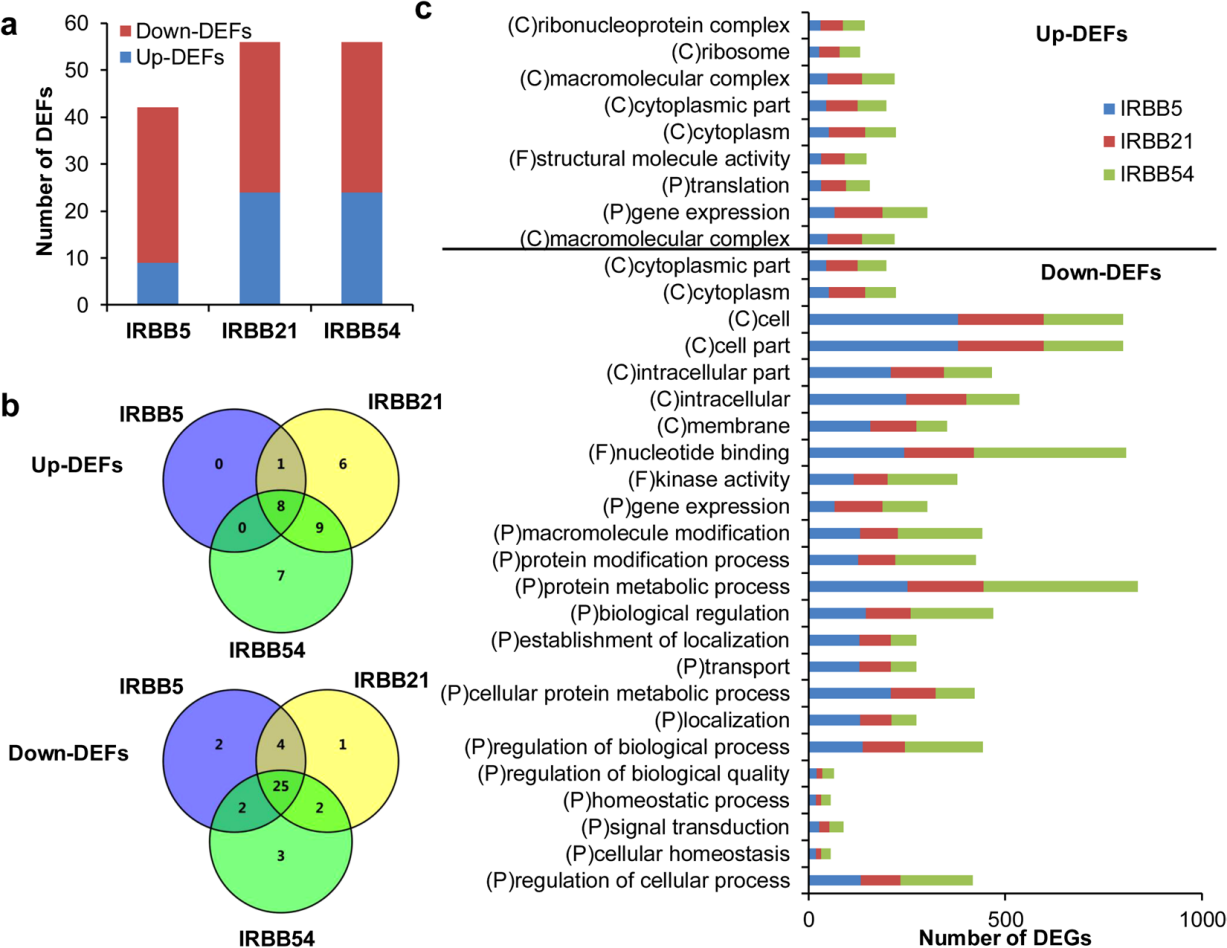
Up- and down-DEFs in the three resistant NILs. The GO functions of DEGs were defined as DEFs. (**a**) The number of up- and down-DEFs in the three resistant NILs; (**b**) Venn diagram of up- and down-DEFs; (**c**) The common up- and down-DEFs in the three resistant NILs. P, biological process; C, cellular component; F, molecular function.

The GO functions of the upregulated and downregulated DEGs were analyzed separately^26^. For convenience, the up-/downregulated DEGs were defined as up-/down-DEGs and the enriched GO functions of up-/down-DEGs were defined as up-/down-DEFs. The up- and down-DEGs were assigned to three GO classes: biological process, cellular component, and molecular function. The up-DEFs and down-DEFs in the three resistant NILs can be found as Supplementary Table S2. As shown in Fig. 2a, the number of down-DEGs in the three resistant NILs was higher than the number of up-DEGs. We found that the number of down-DEFs in the three resistant NILs was also higher than the number of up-DEFs, e.g., 33 down-DEFs vs. 9 up-DEFs in IRBB5, and 32 down-DEFs vs. 24 up-DEFs in both IRBB21 and IRBB54 (Fig. 3a, Supplementary Table S2). Interestingly, we found that 100% (9 vs. 9) of the up-DEFs and 90.6% (29 vs. 32) of the down-DEFs in IRBB5 overlapped with the up-DEFs and down-DEFs in IRBB21. Moreover, 88.9% (8 vs. 9) and 70.8% (17 vs. 24) of the up-DEFs in IRBB5 and IRBB21, respectively, overlapped with the 24 up-DEFs detected in IRBB54 (Fig. 3b, Supplementary Table S2). The down-DEFs in the pyramided line had an 81.8% (27 vs. 33) and 84.4% (27 vs. 32) overlap with the down-DEFs in IRBB5 and IRBB21, respectively (Fig. 3b, Supplementary Table S2). These results suggested that the *Xa21* gene adopted most of the BB resistance characteristics derived from the *xa5* gene and most of the BB resistance characteristics derived from *xa5* and *Xa21* were transmitted to, and pyramided in, IRBB54. On the other hand, eight novel up-DEFs and 3 down-DEFs were observed in the pyramided line, suggesting that novel BB resistance mechanisms may be generated through interaction between *xa5* and *Xa21* (Fig. 3b, Supplementary Table S2).

Additionally, we noticed that 8 up-DEFs and 25 down-DEFs were common among the three resistant NILs (Fig. 3b). Only one up-DEF (structural molecule activity) and two down-DEFs (kinase activity and nucleotide binding) were assigned to the molecular function GO class; most of the common DEFs were assigned to the other two GO classes, indicating that the R genes mainly regulate genes associated with cellular components and biological processes (Fig. 3c).

### Expression patterns and functions of common DEGs

The pyramided line was superior to either of its donor parents for BB resistance. Therefore, the gene expression pattern of the pyramided line is of interest. DEGs that showed differential expression not only between resistant and susceptible lines, but also between any two resistant NILs, were used to determine the gene expression pattern in the pyramiding line (see methods). Surprisingly, an additive expression pattern, which is often used to explain heterosis in hybrids^27^, was not observed in the pyramided line (Table 1). Instead, 97 (77.6%) of DEGs were *Xa21*-dominant (Table 1), i.e., the expression level in IRBB54 was similar to IRBB21 and only 6 (4.8%) of DEGs had expression levels similar to IRBB5 (Table 1). The expression patterns of the other 7 (5.6%) and 15 (12.0%) DEGs showed underdominance and overdominance, respectively (Table 1).

**Table 1:** Distribution of the expression patterns of common differentially expressed genes.

Expression pattern	Genetic class	Number	Sub-total	Ratio	Sub-total
Additive	IRBB5 > IRBB54 > IRBB21	0	0	0.00%	0.00%
	IRBB21 > IRBB54 > IRBB5	0		0.00%	
*xa5*-dominant	IRBB5 = IRBB54 > IRBB21	3	6	2.40%	4.80%
	IRBB5 = IRBB54 < IRBB21	3		2.40%	
*Xa21*-dominant	IRBB5 < IRBB54 = IRBB21	66	97	52.80%	77.60%
	IRBB5 > IRBB54 = IRBB21	31		24.80%	
Under-dominance	IRBB5 > IRBB21 > IRBB54	2	7	1.60%	5.60%
	IRBB5 = IRBB21 > IRBB54	5		4.00%	
	IRBB21 > IRBB5 > IRBB54	0		0.00%	
Over-dominance	IRBB54 > IRBB5 > IRBB21	0	15	0.00%	12.00%
	IRBB54 > IRBB21 > IRBB5	1		0.80%	
	IRBB54 > IRBB21 = IRBB5	14		11.20%	
Total		125	125	100.00%	100.00%

**Figure 4:**
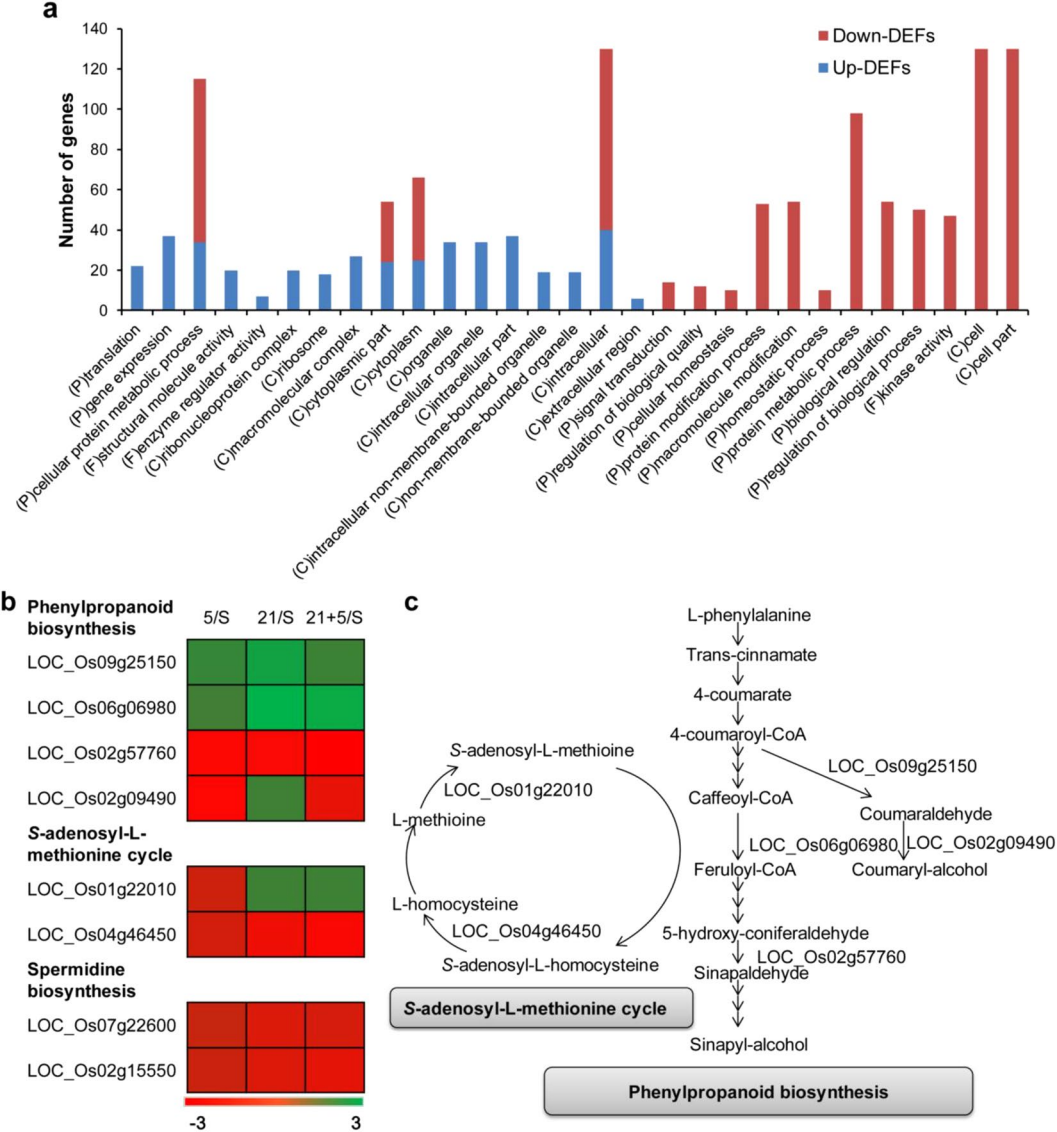
Functional enrichment of common DEGs. (**a**) Enriched GO terms of the common DEGs. P, biological process; C, cellular component; F, molecular function. (**b**) Expression patterns of detected DEGs that are involved in phenylpropanoid biosynthesis, the S-adenosyl-L-methionine (SAM) cycle, and spermidine biosynthesis. (**c**) Reaction step in the biosynthesis pathway in which each of the detected DEGs in (**b**) is involved.

Of the 847 common DEGs identified in the three resistant NILs, 222 (26.2%) were commonly upregulated and 612 (72.3%) were commonly downregulated (Supplementary Fig. S3). The GO terms of the common up-DEGs and down-DEGs were also unexpected. The 222 up-DEGs were significantly enriched in 17 GO terms, whereas the 612 down-DEGs were significantly enriched in 16 GO terms (Supplementary Table S3). Only four GO terms, comprising cellular protein metabolic process, cytoplasmic part, cytoplasm, and intracellular, were common between up- and down-DEFs. Most of the up-DEFs (12 vs. 17) were assigned to the cellular component and most of the down-DEFs (10 vs. 16) were assigned to biological process in the GO class, indicating that they may be involved in basic but durable resistance to BB (Fig. 4a, Supplementary Table S3).

Three pathways were enriched in the common DEGs, including phenylpropanoid biosynthesis, *S*-adenosyl-l-methionine (SAM) cycle, and spermidine biosynthesis (Fig. 4b,c). The expression levels of these common DEGs in the enriched pathways were differentially regulated in the three resistant NILs (Fig. 4b,c), suggesting that they might play different roles in different R gene-mediated resistance responses.

### Effects of gene pyramiding on agronomic traits

Constitutive expression of *xa5* and *Xa21* may have deleterious effects on host growth, especially in the pyramided line that possesses a large number of DEGs. Among the 2,367, 2,412, and 3,596 DEGs in IRBB5, IRBB21, and IRBB54, 365 (98 upregulated and 267 downregulated), 372 (147 upregulated and 225 downregulated) and 551 (146 upregulated and 405 downregulated) genes, respectively, had the R-motif typically found in the 5′ leader sequence of genes with altered translational efficiency during plant immune responses^28^ (Dataset 1). When we investigated further the GO functions of all these up-DEGs and down-DEGs, we found that in the three resistant NILs, the down-DEGs were significantly enriched in GO terms, e.g., 14 in IRBB5, 17 in IRBB21, and 15 in IRBB54, whereas only the up-DEGs in IRBB21 were enriched in 3 GO terms. The common enriched GO terms of the three down-DEGs lists were belong to the biological process GO class (Supplementary Table S4). These results suggested that the disease-resistance genes tend to suppress the function of genes that regulate basic biological process.

To further analyze potential side-effects of disease resistance on the important agronomic traits of hosts, we analyzed the expression levels of 223 curated agronomic trait-controlled genes (downloaded from http://www.ricedata.cn/gene/) in the three resistant NILs (Supplementary Table S5). These 223 genes included 33 genes that function in plant architecture, 50 genes for leaf color and leaf shape, 5 genes for leaf aging and necrosis, 2 genes for leaf inclination, 5 genes for flowering phase, 9 genes for pollen development, 19 genes for floral organ development, 21 genes for heading date, 4 genes for spike sprouting, 14 genes for spike shape, 2 genes for cold tolerance of seed germination, 5 genes for seed shattering, 22 genes for grain shape and grain weight, 2 genes for grain number per panicle, and 8 genes for grain quality. We found that 23, 17, and 34 genes that controlled plant architecture, leaf color and leaf shape, flowering, floral development, male sterility, heading date, grain number, seed shattering, low-temperature germinability, grain quality, and fragrance were differentially expressed in IRBB5, IRBB21 and IRBB54, respectively (Table 2). However, the expression of 73.9% (17 vs. 23), 76.5% (13 vs. 17), and 85.3% (29 vs. 34) of the DEGs was downregulated in IRBB5, IRBB21 and IRBB54, respectively (Table 2). More importantly, only 3 (13.0%), 3 (17.6%) and 5 (14.7%) of the DEGs in IRBB5, IRBB21 and IRBB54, respectively, had R-motifs in their 5′ leader sequence, indicating that side-effects from gene pyramiding on the agronomic traits of plants are minor (Table 2).

**Table 2:** Differentially expressed genes related to agricultural traits.

Trait	Gene symbol	MSU_Locus	Log_2_Fold change		
			IRBB5 vs.IR24	IRBB21 vs.IR24	IRBB54 vs. IR24
Plant architecture	d61; OsBRI1	LOC_Os01g52050			−1.09138
	d35; OsKO2; OsKOL2; OsKOS3	LOC_Os06g37364	5.19089		3.82001
	d18; OsGA3ox2	LOC_Os01g08220	−1.15146		
	d50;5PTase	LOC_Os02g27620*		−1.88278	−2.45244
	D53	LOC_Os11g01330			−1.32443
	sdt; OsmiR156h	LOC_Os06g44034	−2.62083	−2.4453	−2.57991
	DGL1	LOC_Os01g49000	−1.03082	−2.39966	−1.55808
	OsDWARF4; CYP90B2	LOC_Os03g12660	1.19424	1.60517	
Leaf color and leaf shape	WSL12; OsNDPK2	LOC_Os12g36194	−2.45542	−2.23626	−3.42447
	WSP1	LOC_Os04g51280			−1.23409
	ASL2; RPL21c	LOC_Os02g15900	1.04275	1.06502	
	Se5; OsHY1; OsHO1; ygl2; grc1	LOC_Os06g40080		−1.19716	
	YL1	LOC_Os02g05890	−1.31067	−1.54862	−1.24634
	BGL11(t)	LOC_Os11g38040	−2.77189	−2.68554	−2.95883
	NYC1	LOC_Os01g12710			−1.69558
	NOL	LOC_Os03g45194	−1.50762	−1.4619	−2.24912
	YLC1; OsV5A	LOC_Os09g21250	−1.1087	−2.32767	−2.74288
	NAL9; VYL; ClpP	LOC_Os03g29810			−1.47524
	SPL28	LOC_Os01g50770		−2.01764	−2.6691
	OsHsfA4d; Spl7	LOC_Os05g45410	−1.39258		−1.2356
	spl5; SF3b3; 0sSL5	LOC_Os07g10390			−1.0955
	SPL3; OsEDR1; OsACDR1; OsMAPKKK1	LOC_Os03g06410	−1.55476	−1.37346	
	SRL2; AVB; NRL2	LOC_Os03g19520*	−1.5701		
	RL14	LOC_Os10g40960	−1.48141		
	ACL2	LOC_Os02g33330		3.65825	1.94451
Leaf aging and necrosis	OsNaPRT1; LTS1	LOC_Os03g62110			−1.93727
	NLS1	LOC_Os11g14380*			−1.47394
Flowering phase	OsFKF1	LOC_Os11g34460*		−1.3657	
	OsCO3	LOC_Os09g06464*			−1.29873
Pollen sterility	COX11	LOC_Os03g50940	−1.14302		
	UbL404	LOC_Os09g31031			2.70476
Floral organ development	FON1	LOC_Os06g50340	2.48489		
	OsMADS1; LHS1; AFO	LOC_Os03g11614*	−2.90314	−3.40898	−3.08375
Heading date	SDG724; lvp1; OsSET34	LOC_Os09g13740			−2.66602
	Hd17; Ef7; OsELF3; OsELF3-1; OsELF3.1; Hd-q	LOC_Os06g05060			−1.02506
	Hd6; CK2α	LOC_Os03g55389*			−1.39385
	Hd16; CKI; EL1	LOC_Os03g57940			−1.28229
Spike sprouting	β-OsLCY; zebra524	LOC_Os02g09750			−1.2212
	OsPDS	LOC_Os03g08570			−1.32846
Spike shape	LP; EP3	LOC_Os02g15950			−1.37519
Seed shattering	qSH1	LOC_Os01g62920*	1.4227		
Grain shape and grain weight	SRS5; TID1	LOC_Os11g14220	−1.20487		
	D2; CYP90D2; smg11	LOC_Os01g10040			1.84531
	GS5	LOC_Os05g06660	−1.90103		−2.62763
Grain number per panicle	Gn1a; OsCKX2	LOC_Os01g10110			−1.3632
Fragrance	BADH2 (fgr)	LOC_Os08g32870	1.03846	1.67364	
Low-temperature germinability	qLTG3-1	LOC_Os03g01320	−1.40969		1.02667

Pleiotropic genes list only one trait they control. Genes that have the R-motif within their 5′ leader sequence, a typical motif of genes with altered translation during plant immune responses, were marked with*.

## Discussion

The exploration of gene function is at the core of the post-genomic era. NILs, which ideally have identical genetic backgrounds except for the target gene, provide an ideal system to study the function of a gene of interest, where any functional or phenotypic difference between two NILs can be attributed to the target gene.

A simple way to create NILs is backcrossing, followed by selection toward the target phenotype. Indeed, backcrossing may be the only option for creating NILs when the target gene has not been cloned. For genomic regions unlinked to the target gene, backcrossing can rapidly eliminate different genetic backgrounds; however, backcrossing is less efficient for genomic regions linked to target genes. If the target gene has been cloned, transgene technology can be a better option to create NILs with more similar genetic backgrounds, helping to avoid linkage drag in backcrossing. For example, our previous study revealed that the introduction of the *Xa21* gene into rice plants by transgene technology resulted in substantially fewer DEGs than by backcrossing^29^. However, the integration site of a target gene is uncontrollable in transgene technology, complicating analysis of target gene function. However, the recent advent of the CRISPR/Cas9 system has facilitated the accurate and high-throughput editing of target genes *in situ*, thus avoiding complications associated with random integration^30,31^; for example, a herbicide-resistant rice variety was developed recently by editing a single base^32^. Nevertheless, tissue culture procedures are necessary for both the transgene technique and CRISPR/Cas9 system and random mutations in the host genome are common during tissue culture, which hampers the analysis of target gene function. Under such scenarios, traditional backcrossing can be used after the transgene or gene editing procedure to eliminate the mutations among NILs.

The inoculation experiments showed that the rice line harboring both the *Xa21* and *xa5* genes exhibited a stronger level of resistance, and wider resistance spectrum, to *Xoo* strains than the lines with a single R gene, suggesting that there is a positive interaction between the two R genes. However, the combination of *xa5* with *Xa27*, *Xa10*, or *Xa23* failed to promote BB resistance^7,17,18^, highlighting the need to understand the underlying molecular mechanisms of R gene pyramiding so as to predetermine its validity. *AvrXa27*, *avrXa10*, and *avrXa23*, the bacterial avirulence (*avr*) genes of *Xa27*, *Xa10*, and *Xa23*, respectively, are transcription activator-like (TAL) effectors^7,12,33^. The dominant *Xa5* gene, coding for the basal transcription factor TFIIA gamma subunit, is a nuclear target of several bacterial TAL effectors^18^, suggesting that *Xa5* might play a role in the resistance expression of *Xa27*, *Xa10*, and *Xa23*. When *Xa5* is replaced with a recessive *xa5*, *Xa27*, *Xa10*, and *Xa23* may not be activated in the pyramided lines of *xa5*+ *Xa27*/*Xa10/Xa23*, resulting in the invalidation of the R gene combinations. Unlike *Xa27*, *Xa10*, and *Xa23*, the expression of *Xa21* is constitutive^25^ (Fig. 1b) and independent of *Xa5*. Therefore, it is not surprising to observe that the DEGs and DEFs from *xa5* and *Xa21* were pyramided in IRBB54 (Figs 2b and 3b), which suggestted the pyramiding of resistance mechanisms from the two R genes and subsequent enhanced BB resistance in the pyramided line, which was observed in this (Fig. 1d) and previous studies^15^. As such, the pyramiding of independent R genes is expected to be effective; alternatively, a case-by-case analysis may be needed to predict the changes to resistance mechanisms for pyramiding of inter-dependent R genes.

As previously reported, *Xa21* is a dominantly inherited R gene that confers wide-spectrum resistance to BB^34^. Our previous research also showed that rice lines into which *Xa21* was introduced by transgene technology or backcrossing were highly resistant to nine Philippine *Xoo* strains, including the P8 strain^29^. However, the rice line IRBB21, harboring the *Xa21* gene, was only moderately resistant to most of the *Xoo* strains used in this study (Figs 1d and S2), and the P8 strain fully overcame the resistance conferred by *Xa21* (Figs 1d and S2). Since its discovery, *Xa21* has been widely deployed in breeding programs to control rice BB, and the attenuation of resistance observed in this study can be attributed to the long-term co-evolution of *Xoo* and rice cultivars. Recent studies on the *Xa21*-mediated immune response have provided a possible molecular mechanism for the loss of *Xa21*-conferred resistance. The bacterial Rax proteins, including RaxA, RaxB, and RaxC^35^, are required for the activation of *Xa21* and are predicted to comprise a type I secretion system (T1SS). *Xa21* activation by Rax proteins occurs when RaxX is sulfated by RaxST and is then secreted from the bacterial cell by the RaxABC T1SS^36^. The P6 *Xoo* strain harboring either an *raxX* deletion or *raxST* mutation can evade the *Xa21*-mediated immune response^35^. Contrary to previous observations^29^, the P8 strain in this study not only overcame *Xa21*-mediated resistance, but also induced the longest lesions in the susceptible line IR24 (Fig. 1d, Supplementary Fig. S2), suggesting that the broad resistance of *Xa21* has been partially overcome during host-pathogen co-evolution.

It is tempting to speculate that the 847 DEGs common in the three resistant NILs play important roles in rice BB resistance. Of the 847 common DEGs, 8 were expressed only in the resistant NILs or the susceptible line IR24 (Dataset 1) and two of these are seemingly related to rice BB resistance: UDP-glucuronosyl/UDP-glucosyl transferase (LOC_Os05g42060), which is involved in the biosynthesis of cytokinins, phytohormones that play an important role in *Xa21*-mediated BB resistance^37^; and OsWAK receptor-like protein kinase (LOC_Os02g42190), which functions in stress/defense signal perception and transduction, and is adopted by the *Xa21*-mediated resistance pathway^38^. Additionally, the common DEGs are enriched in the phenylpropanoid biosynthesis pathway, *S*-adenosyl-l-methionine (SAM) cycle, and spermidine biosynthesis pathway. The phenylpropanoid-derived metabolites flavonoid, lignin, suberin, and condensed tannins are involved in plant growth, development, and defense^39^; the genes for SAM hydrolase (SAHH) and SAM synthase (SAMS), which function in the SAM cycle, are induced by fungal elicitor^40^; SAHH, which has been reported to play an important role in plant biotic and abiotic stress responses^41^; and Spermidine, a polyamine that is reportedly involved in stress responses and stress tolerance^42^. These results suggested that the SAM cycle, phenylpropanoid metabolites, and spermidine played a role in resistance pathways mediated by rice BB R genes.

We identified 2,367, 2,412, and 3,596 DEGs in IRBB5, IRBB21 and IRBB54, respectively, suggesting broad disturbances at the transcriptional level due to R gene activity (Fig. 2a). Disease resistance is energy-costly, and often at the expense of plant fitness^43,44^. *xa5* and *Xa21* are constitutively expressed throughout the life of the plant, which is an energy-consuming BB resistance tactic. Therefore, these genes might have deleterious effects on normal growth, especially in the pyramided line that exhibited a large number of DEGs. When we performed a deeper analysis into the expression and function of the large numbers of DEGs in the three resistant NILs, we found that most of the DEGs were downregulated and functioned in basal cellular components and biological processes; they were not directly related to stress, suggesting that the R genes tended to suppress basic energy and cellular metabolites to save energy. A recent study showed that genes with an R-motif in the 5′ leader sequence typically exhibited altered translational efficiency during plant immune responses^28^. Consequently, we looked for the 5′ leader sequence in all the DEGs and 223 curated agronomic trait-controlled genes in the three resistant NILs. The discovery of only a limited number of DEGs with an R-motif suggested that the side-effects of plant resistance on rice agronomic traits may not be as great as those suggested by the transcriptome data (Table 2). Moreover, the latest research on microbe-associated molecular pattern-triggered immunity found that engineered plant R genes with translation regulators allowed for plant disease resistance without costs to fitness^45^. With this method, engineered constitutively-expressed R genes could be widely used in future breeding programs.

## Methods

### Plant materials

The susceptible rice line IR24 and three resistant NILs (IRBB5, IRBB21, and IRBB54) were used in this study. IRBB5 and IRBB21 were bred by introducing *Xa21* and *xa5* into IR24 through more than six generations of backcrossing and resistance selection^16,46^. IRBB54 was developed by crossing IRBB5 with IRBB21 and using marker-assisted selection toward plants with both *xa5* and *Xa21* genes^15^.

### Genetic background analysis

Genomic DNA was isolated from fresh rice leaf tissue using the cetyltrimethyl ammonium bromide protocol. The integration of *Xa21* and *xa5* genes in the resistant NILs was validated by polymerase chain reaction (PCR) based on molecular markers UI and I2, and xa5/XhoIF and xa5/XhoIR, respectively (Supplementary Table S6). The genetic background analysis of the three resistant NILs with respect to susceptible IR24 were performed based on SNPs derived from transcriptome data and SSRs derived from the AmpSeq-SSR genotyping data^22^. For SNP genotyping, all the sequence reads were first aligned with the *Japonica* reference genome (irgsp1.0) with Bowtie2 (version 2.1.0) and effective SNPs were identified using SAMtools mpileup (version 1.2) and BCFtools (version 1.3.1) with default parameters. To achieve high accuracy in SNP calling, only consistent SNP sites between the two replicates of each rice line were kept and potential differences in genetic backgrounds between each NILs and IR24 were estimated based on these highly reliable SNPs. AmpSeq-SSR genotyping, which combined super multiplex-PCR and high-throughput sequencing, was also used to calculate the similarity in the genetic backgrounds of the resistant NILs^22^. Details for AmpSeq-SSR genotyping can be seen in the authors’ previous report^22^. Libraries for AmpSeq-SSR genotyping were constructed according to the user guide for the Ion AmpliSeq™ Library Kit 2.0 (CatNo. 4475345, Thermo Fisher Scientific, Waltham, MA, USA). 3105 SSRs, including forty-eight SSRs that are listed in the National Agricultural Standard of China (NY/T 1433–3014), and 3057 randomly selected SSRs from the *Japonica* reference genome (irgsp1.0) were chosen as target SSRs. The primers for target SSRs were designed at https://ampliseq.com/ and synthesized by Thermo Company, USA. The full list of primers has been reported previously^22^. All primers were pooled and 14 PCR cycles were performed for DNA amplification. The resulting libraries were sequenced on the Ion S5™ next-generation sequencing system (Cat. No. A27212, Thermo Fisher Scientific, Waltham, MA, USA) using single-end sequencing with a length of 300 bp. Strict quality control was conducted for the raw reads of each sample. All reads shorter than 100 bp and that could not be mapped to the targeted regions were discarded. Moreover, only SSRs with a coverage of at least 20 reads and a stutter ratio no greater than 0.5 were regarded as valid SSRs^22^. The genotype represented by the most number of reads is recorded as the major genotype of the SSR locus and the stutter ratio of the SSR locus is the ratio between the number of reads of the second genotype and major genotype. Based on the results of SSR genotyping, all sites consistent between IR24 and each NIL were recorded as comparable SSRs and used to compare genetic backgrounds.

### *Xoo* cultivation, inoculation, and analysis of resistance level

Eight representative *Xoo* strains from the Philippines, including P1 (PXO61), P2 (PXO86), P3 (PXO79), P4 (PXO71), P6 (PXO99), P7 (PXO145), P8 (PXO280), and P10 (PXO341) were used in this study. Each *Xoo* strain was suspended in sterile water at a concentration of 10^9^ cells/ml and inoculated at similar positions on three to five leaves using the leaf clipping method^47^ at the maximum tillering stage. Lesion lengths of 10 inoculated leaves from each tested rice line were measured 15 days after inoculation. Lesion lengths of ≤3 cm, 3–6 cm, 6–10 cm, and ≥10 cm were respectively determined as resistant (R), moderately resistant (MR), moderately susceptible (MS), and susceptible (S) based on the standard disease rating system for lesion length^48^.

### Library preparation and high-throughput sequencing

Ten leaves were randomly harvested from 10 individuals of each rice line and pooled for RNA extraction. Total RNA was isolated using TRIzol reagent (Invitrogen, Carlsbad, CA, USA) following the manufacturer’s instructions. The RNA integrity number was evaluated with an Agilent^®^ 2100 Bioanalyzer^®^ instrument. Only RNA with an integrity number >7 was used for library construction. mRNA was purified from 20 μg of total RNA using the NEB next poly(A) mRNA magnetic isolation module (Cat. No. E7490; New England Biolabs, Ipswich, MA, UK). Approximately 100 ng of mRNA was used to construct RNA-seq libraries using the Ion Total RNA-Seq Kit v2 (Cat. No. 4479789, Thermo Fisher Scientific, Waltham, MA, USA) following the manual. Each sample performed two biological replicates. The yield and size distribution of the libraries were assessed with the Agilent^®^ 2100 Bioanalyzer^®^ and Agilent^®^ High Sensitivity DNA Kit (Cat. No. 5067-4626; Agilent Technologies, Santa Clara, CA, USA). Sequencing chips were prepared on the Ion Chef^TM^ system and sequencing was carried out on the Ion S5™ next-generation sequencing system (Cat. No. A27212, Thermo Fisher Scientific, Waltham, MA, USA).

### Differential expression analysis

Quality check was conducted on all raw data. Reads shorter than 50 bp, with adapter sequences, or with poly-N sequences were discarded. The remaining reads were mapped to the rice reference genome (MSU 7.0) using Tophat (version 2.0.13). Cufflinks (version 2.0.2) was used to assemble the mapped reads with default parameters and estimate the expression of each transcript^49^. The number of qualified reads for each gene was normalized to TPM (number of transcripts per million qualified reads), which was then used as the digital gene expression abundance of the gene. The Benjamini & Hochberg method was used to adjust the *P*-value for multiple tests^50^. Significant DEGs across two samples were determined with the *P* value cut-off of less than 0.05 and an absolute value of log_2_ fold change ≥1. DEGs that showed differential expression not only between resistant and susceptible lines, but also between any two resistant NILs, were used to determine gene expression patterns in the pyramided line.

### R-motif analysis

R-motifs within 5′ leader sequences of DEGs were scanned by the online FIMO tool, with default settings, in the MEME suits^51^. The R-motif frequency matrix was provided by Xu^28^.

### Quantitative reverse-transcription (qRT-) PCR

Two micrograms of total RNA was extracted for first-strand cDNA synthesis in a 20-μL reaction volume using M-MLV reverse transcriptase (Promega) and oligo (dT) 18 primer according to the manufacturer’s protocol. The reaction mixture contained 0.3 μL cDNA, 0.2 μM primers (Supplementary Table S4), 10 μL Tran*Start*^®^ TipTop Green qPCR SupMix reagent, and 0.4 μL ROX as a passive reference dye (Cat. No. AQ141; TransGen Biotech. China). The mixture was loaded on the Applied Biosystems StepOne^TM^ Real-Time PCR machine for real-time PCR detection using a procedure of 30 s at 95 °C, 40 cycles of 5 s at 95 °C and 30 s at 60 °C, followed by melting analysis. The relative expression levels of *Xa21* and *xa5* were analyzed by qPCR using IR24 as a reference sample and the rice *ubiquitin* gene as the internal control gene^21^. The primers for the *ubiquitin* gene were synthesized based on a previous study^21^. The 2^−ΔΔCT^ method was used to estimate the relative expression changes of target genes^52^. Three biological replicates were included in this experiment. The primers for qPCR analysis are listed in Supplementary Table S6.

### Gene Ontology and pathway enrichment analysis

Gene Ontology were assigned to DEGs using the web tool agriGO v2.0^53^. Plant GOslim was selected for GO enrichment analysis. A Hypergeomotric test was used to calculate the enrichment of GO terms^26^ and the GO terms with an FDR less than 0.05 after multi-test adjustments by the Yekutieli method (FDR under dependency) were considered significantly enriched^54^. Genes were associated with metabolic pathways using the RiceCyc pathway database (version 3.3, http://pathway.gramene.org/ricecyc.html). Pathways with *P* < 0.05 were considered enriched.

### Data availability

The datasets generated during the current study are available in the GenBank repository https://trace.ncbi.nlm.nih.gov/Traces/sra_sub/sub.cgi?acc=SRP108493&focus=SRP108493&from=submission&action=show:STUDY. The datasets will be publicly available upon acceptance of the manuscript.

## Electronic supplementary material

Supplemental legends

Supplementary figures and tables

Dataset 1
