# Supplementary material for: Transcriptional insights into the pyramided resistance to rice bacterial blight
Source: Sci Rep. 2018 Aug 17;8:12358. doi: 10.1038/s41598-018-29899-1 (PMC6098014; doi:10.1038/s41598-018-29899-1)
Supplement: Supplementary file 1 — Supplemental legends [file 41598_2018_29899_MOESM1_ESM.docx]

Transcriptional insights into the pyramided resistance to rice bacterial blight

Lifen Gao^1, +^, Zhiwei Fang^1, +^, Junfei Zhou^1^, Lun Li^1^, Long Lu^1^, Lili Li^1^, Tiantian Li^1^, Lihong Chen^1^, Weixiong Zhang^1^, Wenxue Zhai^2, *^ and Hai Peng^1, *^

^1^Institute for Systems Biology, Jianghan University, Wuhan, Hubei 430056, China;

^2^Institute of Genetics and Developmental Biology, Chinese Academy of Sciences, Beijing 100101, China;

^+^These authors contributed equally to this work

^*^Corresponding author: Hai Peng (penghai@jhun.edu.cn) and Wenxue Zhai (wxzhai@genetics.ac.cn)

**Supplemental figures legends**

**Figure S1. Genetic background analysis of the three resistant NILs by SNPs.** (a) The distribution of SNPs on chromosome 5. (b) The distribution of SNPs on chromosome 11. The black arrows indicate the chromosome location of *xa5* (a) and *Xa21* (b).

**Figure S2. Phenotype of IR24, IRBB21, IRBB5 and IRBB54 after inoculation with eight *Xoo* strains.** P1-P10: *Xoo* strains.

**Figure S3. Venn diagram of common up- and down-DEGs in the three resistant NILs.** The up- and down DEGs represent the up-regulated and down-regulated DEGs, respectively.

**Figure S4. The full-length gels for Fig. 1a.** The other rice lines were not included in this study

**Supplemental Tables legends**

**Table S1. Differential SSRs in IRBB5, IRBB21 and IRBB54 with respect to IR24.** *represent the SSRs showed different genotypes between two samples.

**Table S2.** **Up-DEFs and down-DEFs in IRBB5, IRBB21 and IRBB54.** The up-DEFs and down-DEFs were the significant enriched GO terms of up-regulated DEGs and down-regulated DEGs respectively. The P, F and C in the ontology column were on behalf of biological process, molecular function and cellular component, respectively.

**Table S3. Up-DEFs and down-DEFs enriched by the common up- and down-DEGs in IRBB5, IRBB21 and IRBB54.** The up-DEFs and down-DEFs represented the significant enriched GO terms of common up- and down-DEGs in IRBB5, IRBB21 and IRBB54 respectively. The P, F and C in the ontology column were on behalf of biological process, molecular function and cellular component respectively.

**Table S4. Up-DEFs and down-DEFs enriched by the DEGs with R-motif in IRBB5, IRBB21 and IRBB54.** *indicate the common enriched GO terms among the three down-regulated DEGs list. The up-DEFs and down-DEFs were the significant enriched GO terms of up-DEGs and down-DEGs respectively. The P, F and C in the ontology column were on behalf of the GO ontology biological process, molecular function and cellular component respectively.

**Table S5. The 223 curated agronomic traits-controlled genes used for the side effects analysis of gene pyramiding.** Pleiotropic genes list only one trait they controls. Genes that have R-motif within their 5’ leader sequence, a typical motif of genes with altered translation during plant immune response, were marked with *.

**Table S6. Primers used for detecting the integration and expression of *xa5* and *Xa21*.**

**Dataset 1.** **Total and common DEGs in IRBB5, IRBB21 and IRBB54;** “inf” means the gene has no expression in IRBB5 and “-inf(#name)” means the gene has no expression in IR24. *represent the significant expression difference between two samples. # represent the gene with R-motif in the 5’ leader sequence.
