# Supplementary material for: Transcriptional insights into the pyramided resistance to rice bacterial blight
Source: Sci Rep. 2018 Aug 17;8:12358. doi: 10.1038/s41598-018-29899-1 (PMC6098014; doi:10.1038/s41598-018-29899-1)
Supplement: Supplementary file 2 — Supplementary figures and tables [file 41598_2018_29899_MOESM2_ESM.docx]

Transcriptional insights into the pyramided resistance to rice bacterial blight

Lifen Gao^1, +^, Zhiwei Fang^1, +^, Junfei Zhou^1^, Lun Li^1^, Long Lu^1^, Lili Li^1^, Tiantian Li^1^, Lihong Chen^1^, Weixiong Zhang^1^, Wenxue Zhai^2, *^ and Hai Peng^1, *^

^1^Institute for Systems Biology, Jianghan University, Wuhan, Hubei 430056, China;

^2^Institute of Genetics and Developmental Biology, Chinese Academy of Sciences, Beijing 100101, China;

^+^These authors contributed equally to this work

^*^Corresponding author: Hai Peng (penghai@jhun.edu.cn) and Wenxue Zhai (wxzhai@genetics.ac.cn)

**
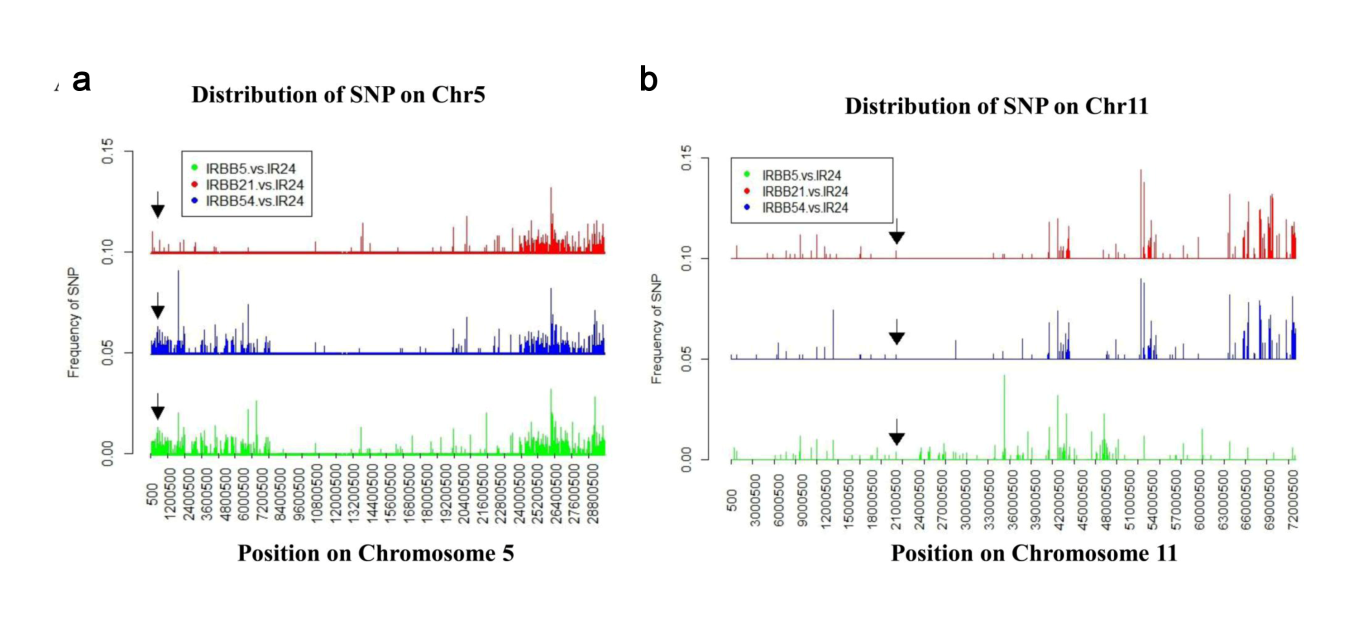
**

**Figure S1. Genetic background analysis of the three resistant NILs by SNPs.** (a) The distribution of SNPs on chromosome 5. (b) The distribution of SNPs on chromosome 11. The black arrows indicate the chromosome location of *xa5* (a) and *Xa21* (b).


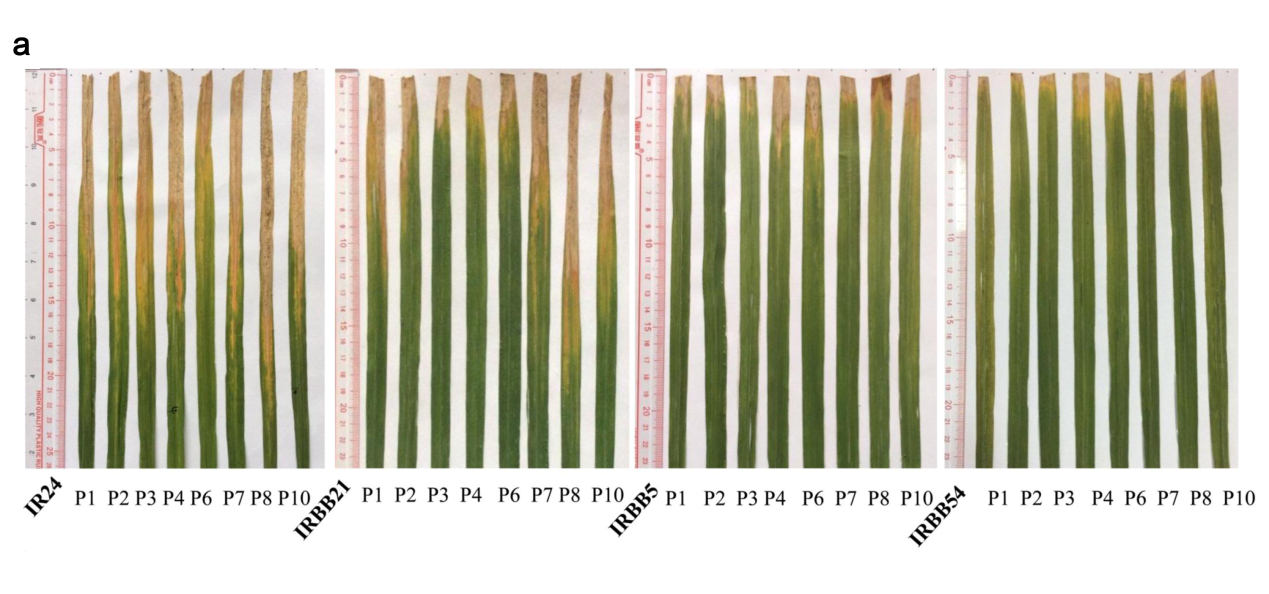


**Figure S2. Phenotype of IR24, IRBB21, IRBB5 and IRBB54 after inoculation with eight *Xoo* strains.** P1-P10: *Xoo* strains.


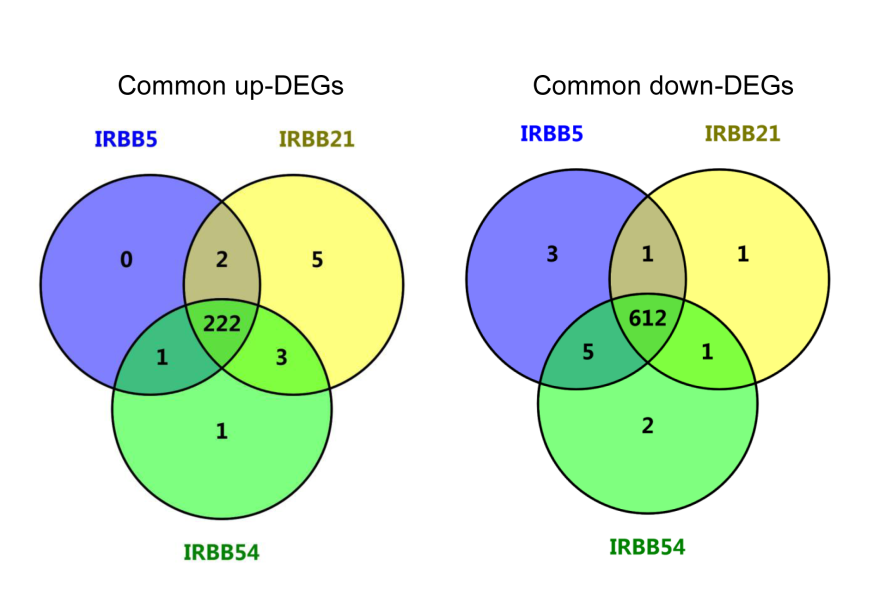


**Figure S3. Venn diagram of common up- and down-DEGs in the three resistant NILs.** The up- and down DEGs represent the up-regulated and down-regulated DEGs, respectively.


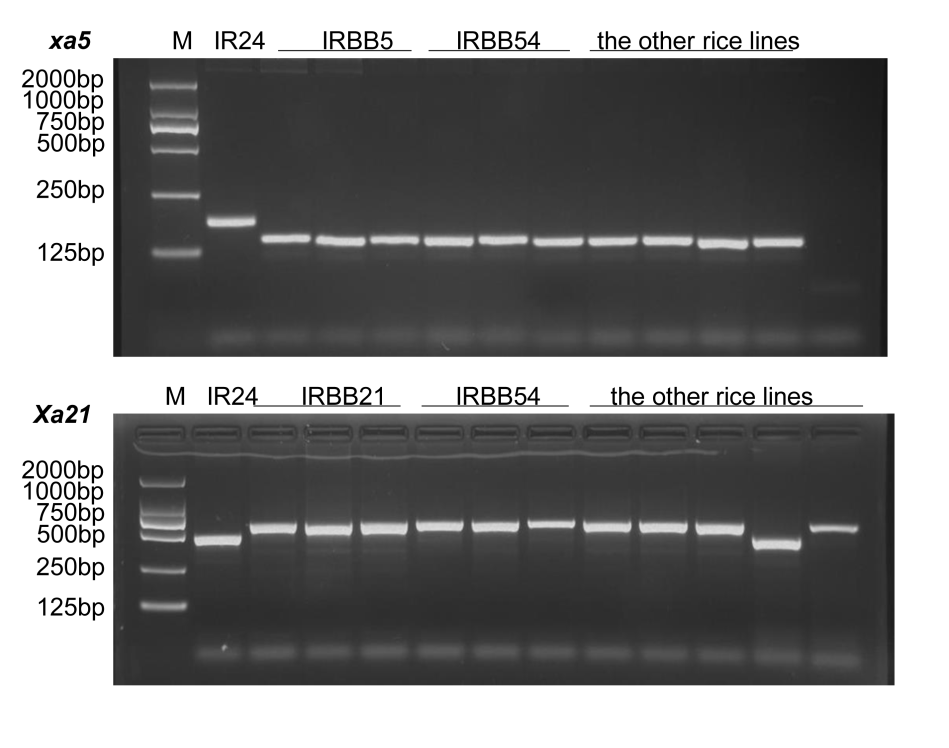


**Figure S4. The full-length gels for Fig. 1a.** The other rice lines were not included in this study

Table S1. Differential SSRs in IRBB5, IRBB21 and IRBB54 with respect to IR24.

| Chromosome | Start position | Stop position | IRBB5 vs. IR24 | IRBB21 vs. IR24 | IRBB54 vs. IR24 |
| --- | --- | --- | --- | --- | --- |
| chr1 | 25368462 | 25368704 | * |  |  |
| chr1 | 24153859 | 24154130 | * |  |  |
| chr2 | 26646357 | 26646631 | * |  |  |
| chr3 | 3069535 | 3069774 | * |  | * |
| chr5 | 26344198 | 26344399 | * |  |  |
| chr5 | 28101312 | 28101586 | * |  | * |
| chr5 | 26136430 | 26136704 | * |  | * |
| chr6 | 23890502 | 23890776 | * |  | * |
| chr6 | 21616932 | 21617206 | * |  | * |
| chr11 | 13868861 | 13869134 | * |  |  |
| chr11 | 15349870 | 15350090 | * |  |  |
| chr2 | 8760307 | 8760581 |  | * |  |
| chr2 | 11068644 | 11068918 |  | * |  |
| chr2 | 11389710 | 11389984 |  | * |  |
| chr2 | 11466523 | 11466797 |  | * |  |
| chr3 | 33157572 | 33157846 |  | * |  |
| chr8 | 6763691 | 6763965 |  | * |  |
| chr8 | 9387060 | 9387333 |  | * |  |
| chr8 | 10523955 | 10524229 |  | * |  |
| chr8 | 15835153 | 15835374 |  | * |  |
| chr8 | 15972649 | 15972923 |  | * |  |
| chr8 | 17540962 | 17541236 |  | * |  |
| chr8 | 17945022 | 17945267 |  | * |  |
| chr11 | 21463811 | 21463982 |  | * | * |
| chr11 | 18274416 | 18274686 |  | * |  |

*represent the SSRs showed different genotypes between two samples

Table S2. The up-DEFs and down-DEFs in IRBB5, IRBB21 and IRBB54

|  | GO term | Ontology | Description | Number of genes | Number in BG/Ref | FDR |
| --- | --- | --- | --- | --- | --- | --- |
| Up-DEFs in IRBB5 | GO:0006412 | P | translation | 32 | 595 | 3.2E-06 |
|  | GO:0010467 | P | gene expression | 67 | 2424 | 0.0036 |
|  | GO:0005975 | P | carbohydrate metabolic process | 28 | 833 | 0.029 |
|  | GO:0005198 | F | structural molecule activity | 33 | 462 | 1.2E-09 |
|  | GO:0030529 | C | ribonucleoprotein complex | 30 | 438 | 3.1E-08 |
|  | GO:0044444 | C | cytoplasmic part | 46 | 966 | 3.1E-08 |
|  | GO:0005840 | C | ribosome | 27 | 397 | 9.2E-08 |
|  | GO:0005737 | C | cytoplasm | 51 | 1263 | 3.5E-07 |
|  | GO:0032991 | C | macromolecular complex | 49 | 1207 | 4.7E-07 |
| Down-DEFs in IRBB5 | GO:0044267 | P | cellular protein metabolic process | 210 | 2877 | 3.1E-07 |
|  | GO:0006810 | P | transport | 130 | 1638 | 2.4E-06 |
|  | GO:0051234 | P | establishment of localization | 130 | 1638 | 2.4E-06 |
|  | GO:0051179 | P | localization | 131 | 1651 | 2.4E-06 |
|  | GO:0010467 | P | gene expression | 164 | 2424 | 0.0004 |
|  | GO:0065007 | P | biological regulation | 146 | 2125 | 0.00053 |
|  | GO:0050789 | P | regulation of biological process | 138 | 2027 | 0.0012 |
|  | GO:0050794 | P | regulation of cellular process | 133 | 1955 | 0.0015 |
|  | GO:0006412 | P | translation | 52 | 595 | 0.0015 |
|  | GO:0007165 | P | signal transduction | 27 | 240 | 0.0022 |
|  | GO:0019725 | P | cellular homeostasis | 19 | 140 | 0.0024 |
|  | GO:0043412 | P | macromolecule modification | 131 | 1979 | 0.0035 |
|  | GO:0042592 | P | homeostatic process | 19 | 147 | 0.0036 |
|  | GO:0065008 | P | regulation of biological quality | 21 | 176 | 0.0043 |
|  | GO:0006464 | P | protein modification process | 126 | 1929 | 0.0061 |
|  | GO:0019538 | P | protein metabolic process | 252 | 4333 | 0.0095 |
|  | GO:0006519 | P | cellular amino acid and derivative metabolic process | 33 | 391 | 0.027 |
|  | GO:0000166 | F | nucleotide binding | 243 | 3874 | 0.00049 |
|  | GO:0005198 | F | structural molecule activity | 46 | 462 | 0.00049 |
|  | GO:0005215 | F | transporter activity | 76 | 941 | 0.00064 |
|  | GO:0016301 | F | kinase activity | 115 | 1750 | 0.016 |
|  | GO:0005737 | C | cytoplasm | 132 | 1263 | 1.5E-13 |
|  | GO:0044444 | C | cytoplasmic part | 95 | 966 | 2.1E-08 |
|  | GO:0044464 | C | cell part | 380 | 5945 | 2.1E-08 |
|  | GO:0005623 | C | cell | 380 | 5945 | 2.1E-08 |
|  | GO:0016020 | C | membrane | 157 | 2115 | 2.7E-06 |
|  | GO:0032991 | C | macromolecular complex | 99 | 1207 | 0.000013 |
|  | GO:0005840 | C | ribosome | 40 | 397 | 0.00055 |
|  | GO:0030529 | C | ribonucleoprotein complex | 42 | 438 | 0.00087 |
|  | GO:0005622 | C | intracellular | 248 | 4116 | 0.0016 |
|  | GO:0005794 | C | Golgi apparatus | 10 | 56 | 0.01 |
|  | GO:0044424 | C | intracellular part | 210 | 3529 | 0.01 |
|  | GO:0005783 | C | endoplasmic reticulum | 14 | 123 | 0.048 |
| Up-DEFs in IRBB21 | GO:0006412 | P | translation | 63 | 595 | 1.7E-14 |
|  | GO:0010467 | P | gene expression | 122 | 2424 | 1.5E-06 |
|  | GO:0006810 | P | transport | 80 | 1638 | 0.00068 |
|  | GO:0051234 | P | establishment of localization | 80 | 1638 | 0.00068 |
|  | GO:0006519 | P | cellular amino acid and derivative metabolic process | 29 | 391 | 0.00068 |
|  | GO:0051179 | P | localization | 80 | 1651 | 0.00074 |
|  | GO:0019748 | P | secondary metabolic process | 9 | 64 | 0.007 |
|  | GO:0005975 | P | carbohydrate metabolic process | 44 | 833 | 0.0076 |
|  | GO:0065008 | P | regulation of biological quality | 15 | 176 | 0.01 |
|  | GO:0019725 | P | cellular homeostasis | 13 | 140 | 0.01 |
|  | GO:0042592 | P | homeostatic process | 13 | 147 | 0.014 |
|  | GO:0009056 | P | catabolic process | 26 | 433 | 0.016 |
|  | GO:0044267 | P | cellular protein metabolic process | 113 | 2877 | 0.026 |
|  | GO:0005198 | F | structural molecule activity | 59 | 462 | 3E-17 |
|  | GO:0008135 | F | translation factor activity, nucleic acid binding | 10 | 75 | 0.0078 |
|  | GO:0030529 | C | ribonucleoprotein complex | 57 | 438 | 6.2E-17 |
|  | GO:0005840 | C | ribosome | 52 | 397 | 8.9E-16 |
|  | GO:0005737 | C | cytoplasm | 94 | 1263 | 1E-13 |
|  | GO:0044444 | C | cytoplasmic part | 79 | 966 | 1.6E-13 |
|  | GO:0032991 | C | macromolecular complex | 87 | 1207 | 4.1E-12 |
|  | GO:0044464 | C | cell part | 218 | 5945 | 0.0057 |
|  | GO:0005623 | C | cell | 218 | 5945 | 0.0057 |
|  | GO:0044424 | C | intracellular part | 135 | 3529 | 0.02 |
|  | GO:0005622 | C | intracellular | 153 | 4116 | 0.026 |
| Down-DEFs in IRBB21 | GO:0044267 | P | cellular protein metabolic process | 168 | 2877 | 1.3E-08 |
|  | GO:0007165 | P | signal transduction | 27 | 240 | 0.000046 |
|  | GO:0010467 | P | gene expression | 131 | 2424 | 0.000056 |
|  | GO:0006412 | P | translation | 45 | 595 | 0.00018 |
|  | GO:0051179 | P | localization | 94 | 1651 | 0.00021 |
|  | GO:0065007 | P | biological regulation | 114 | 2125 | 0.00022 |
|  | GO:0065008 | P | regulation of biological quality | 20 | 176 | 0.00027 |
|  | GO:0006810 | P | transport | 92 | 1638 | 0.00027 |
|  | GO:0051234 | P | establishment of localization | 92 | 1638 | 0.00027 |
|  | GO:0050789 | P | regulation of biological process | 107 | 2027 | 0.00051 |
|  | GO:0042592 | P | homeostatic process | 17 | 147 | 0.00079 |
|  | GO:0050794 | P | regulation of cellular process | 101 | 1955 | 0.0016 |
|  | GO:0019538 | P | protein metabolic process | 193 | 4333 | 0.004 |
|  | GO:0019725 | P | cellular homeostasis | 14 | 140 | 0.01 |
|  | GO:0043412 | P | macromolecule modification | 97 | 1979 | 0.01 |
|  | GO:0006464 | P | protein modification process | 94 | 1929 | 0.013 |
|  | GO:0009056 | P | catabolic process | 28 | 433 | 0.026 |
|  | GO:0005198 | F | structural molecule activity | 38 | 462 | 0.00033 |
|  | GO:0000166 | F | nucleotide binding | 178 | 3874 | 0.0051 |
|  | GO:0016301 | F | kinase activity | 86 | 1750 | 0.044 |
|  | GO:0044464 | C | cell part | 310 | 5945 | 1.1E-12 |
|  | GO:0005623 | C | cell | 310 | 5945 | 1.1E-12 |
|  | GO:0005737 | C | cytoplasm | 95 | 1263 | 6E-10 |
|  | GO:0032991 | C | macromolecular complex | 87 | 1207 | 2.5E-08 |
|  | GO:0005622 | C | intracellular | 213 | 4116 | 7.9E-08 |
|  | GO:0044444 | C | cytoplasmic part | 69 | 966 | 1.5E-06 |
|  | GO:0044424 | C | intracellular part | 179 | 3529 | 0.000006 |
|  | GO:0016020 | C | membrane | 117 | 2115 | 0.000019 |
|  | GO:0005840 | C | ribosome | 32 | 397 | 0.00041 |
|  | GO:0030529 | C | ribonucleoprotein complex | 34 | 438 | 0.00042 |
|  | GO:0012505 | C | endomembrane system | 11 | 104 | 0.021 |
|  | GO:0009536 | C | plastid | 6 | 37 | 0.032 |
| Up-DEFs in IRBB54 | GO:0006412 | P | translation | 61 | 595 | 1E-18 |
|  | GO:0010467 | P | gene expression | 113 | 2424 | 8.9E-11 |
|  | GO:0044267 | P | cellular protein metabolic process | 100 | 2877 | 0.0014 |
|  | GO:0006810 | P | transport | 64 | 1638 | 0.0014 |
|  | GO:0051234 | P | establishment of localization | 64 | 1638 | 0.0014 |
|  | GO:0051179 | P | localization | 64 | 1651 | 0.0015 |
|  | GO:0005198 | F | structural molecule activity | 56 | 462 | 1.8E-20 |
|  | GO:0005215 | F | transporter activity | 43 | 941 | 0.0013 |
|  | GO:0008135 | F | translation factor activity, nucleic acid binding | 8 | 75 | 0.016 |
|  | GO:0030529 | C | ribonucleoprotein complex | 56 | 438 | 1.7E-21 |
|  | GO:0005840 | C | ribosome | 53 | 397 | 2.3E-21 |
|  | GO:0044444 | C | cytoplasmic part | 73 | 966 | 1.3E-16 |
|  | GO:0032991 | C | macromolecular complex | 83 | 1207 | 1.3E-16 |
|  | GO:0005737 | C | cytoplasm | 78 | 1263 | 2.5E-13 |
|  | GO:0044464 | C | cell part | 202 | 5945 | 1.4E-08 |
|  | GO:0005623 | C | cell | 202 | 5945 | 1.4E-08 |
|  | GO:0043229 | C | intracellular organelle | 109 | 2859 | 1.6E-06 |
|  | GO:0043226 | C | organelle | 109 | 2859 | 1.6E-06 |
|  | GO:0043232 | C | intracellular non-membrane-bounded organelle | 66 | 1494 | 5.2E-06 |
|  | GO:0043228 | C | non-membrane-bounded organelle | 66 | 1494 | 5.2E-06 |
|  | GO:0044424 | C | intracellular part | 122 | 3529 | 0.000027 |
|  | GO:0005622 | C | intracellular | 135 | 4116 | 0.000091 |
|  | GO:0016020 | C | membrane | 79 | 2115 | 0.00014 |
|  | GO:0005829 | C | cytosol | 5 | 34 | 0.01 |
| Down-DEFs in IRBB54 | GO:0044267 | P | cellular protein metabolic process | 327 | 2877 | 1.4E-11 |
|  | GO:0006810 | P | transport | 209 | 1638 | 2.5E-11 |
|  | GO:0051234 | P | establishment of localization | 209 | 1638 | 2.5E-11 |
|  | GO:0051179 | P | localization | 211 | 1651 | 2.5E-11 |
|  | GO:0043412 | P | macromolecule modification | 214 | 1979 | 4.9E-06 |
|  | GO:0006464 | P | protein modification process | 206 | 1929 | 0.000017 |
|  | GO:0010467 | P | gene expression | 239 | 2424 | 0.00037 |
|  | GO:0019538 | P | protein metabolic process | 391 | 4333 | 0.001 |
|  | GO:0065007 | P | biological regulation | 209 | 2125 | 0.0013 |
|  | GO:0050789 | P | regulation of biological process | 198 | 2027 | 0.0026 |
|  | GO:0006519 | P | cellular amino acid and derivative metabolic process | 52 | 391 | 0.0029 |
|  | GO:0065008 | P | regulation of biological quality | 29 | 176 | 0.0038 |
|  | GO:0042592 | P | homeostatic process | 25 | 147 | 0.0061 |
|  | GO:0007165 | P | signal transduction | 35 | 240 | 0.0061 |
|  | GO:0019725 | P | cellular homeostasis | 24 | 140 | 0.0062 |
|  | GO:0009056 | P | catabolic process | 54 | 433 | 0.0062 |
|  | GO:0050794 | P | regulation of cellular process | 184 | 1955 | 0.014 |
|  | GO:0000166 | F | nucleotide binding | 387 | 3874 | 2.1E-07 |
|  | GO:0005215 | F | transporter activity | 114 | 941 | 0.000065 |
|  | GO:0016301 | F | kinase activity | 178 | 1750 | 0.0012 |
|  | GO:0005737 | C | cytoplasm | 178 | 1263 | 1.9E-12 |
|  | GO:0044464 | C | cell part | 576 | 5945 | 1.5E-10 |
|  | GO:0005623 | C | cell | 576 | 5945 | 1.5E-10 |
|  | GO:0016020 | C | membrane | 237 | 2115 | 4.3E-08 |
|  | GO:0005622 | C | intracellular | 386 | 4116 | 0.000038 |
|  | GO:0044444 | C | cytoplasmic part | 116 | 966 | 0.000038 |
|  | GO:0009536 | C | plastid | 13 | 37 | 0.00055 |
|  | GO:0044424 | C | intracellular part | 326 | 3529 | 0.00069 |
|  | GO:0005739 | C | mitochondrion | 28 | 162 | 0.0021 |
|  | GO:0032991 | C | macromolecular complex | 127 | 1207 | 0.0021 |
|  | GO:0031967 | C | organelle envelope | 23 | 142 | 0.014 |
|  | GO:0031975 | C | envelope | 25 | 167 | 0.022 |

The up-DEFs and down-DEFs were the significant enriched GO terms of up-regulated DEGs and down-regulated DEGs respectively. The P, F and C in the ontology column were on behalf of biological process, molecular function and cellular component respectively.

Table S3. Up-DEFs and down-DEFs enriched by the common up- and down-DEGs in IRBB5, IRBB21 and IRBB54

|  | GO term | Ontology | Description | Number in input list | Number in BG/Ref | FDR |
| --- | --- | --- | --- | --- | --- | --- |
| Up-DEFs | GO:0006412 | P | translation | 22 | 595 | 5.10E-09 |
|  | GO:0010467 | P | gene expression | 37 | 2424 | 1.80E-05 |
|  | GO:0044267 | P | cellular protein metabolic process | 34 | 2877 | 0.0081 |
|  | GO:0005198 | F | structural molecule activity | 20 | 462 | 1.30E-09 |
|  | GO:0030234 | F | enzyme regulator activity | 7 | 255 | 0.036 |
|  | GO:0030529 | C | ribonucleoprotein complex | 20 | 438 | 7.00E-10 |
|  | GO:0005840 | C | ribosome | 18 | 397 | 4.50E-09 |
|  | GO:0032991 | C | macromolecular complex | 27 | 1207 | 2.10E-07 |
|  | GO:0044444 | C | cytoplasmic part | 24 | 966 | 2.10E-07 |
|  | GO:0005737 | C | cytoplasm | 25 | 1263 | 5.80E-06 |
|  | GO:0043226 | C | organelle | 34 | 2859 | 0.0019 |
|  | GO:0043229 | C | intracellular organelle | 34 | 2859 | 0.0019 |
|  | GO:0044424 | C | intracellular part | 37 | 3529 | 0.0091 |
|  | GO:0043232 | C | intracellular non-membrane-bounded organelle | 19 | 1494 | 0.018 |
|  | GO:0043228 | C | non-membrane-bounded organelle | 19 | 1494 | 0.018 |
|  | GO:0005622 | C | intracellular | 40 | 4116 | 0.018 |
|  | GO:0005576 | C | extracellular region | 6 | 251 | 0.033 |
| Down-DEFs | GO:0044267 | P | cellular protein metabolic process | 81 | 2877 | 0.00011 |
|  | GO:0007165 | P | signal transduction | 14 | 240 | 0.0035 |
|  | GO:0065008 | P | regulation of biological quality | 12 | 176 | 0.0035 |
|  | GO:0019725 | P | cellular homeostasis | 10 | 140 | 0.0042 |
|  | GO:0006464 | P | protein modification process | 53 | 1929 | 0.0042 |
|  | GO:0043412 | P | macromolecule modification | 54 | 1979 | 0.0042 |
|  | GO:0042592 | P | homeostatic process | 10 | 147 | 0.0053 |
|  | GO:0019538 | P | protein metabolic process | 98 | 4333 | 0.0064 |
|  | GO:0065007 | P | biological regulation | 54 | 2125 | 0.014 |
|  | GO:0050789 | P | regulation of biological process | 50 | 2027 | 0.037 |
|  | GO:0016301 | F | kinase activity | 47 | 1750 | 0.043 |
|  | GO:0005737 | C | cytoplasm | 41 | 1263 | 0.002 |
|  | GO:0005623 | C | cell | 130 | 5945 | 0.0022 |
|  | GO:0044464 | C | cell part | 130 | 5945 | 0.0022 |
|  | GO:0044444 | C | cytoplasmic part | 30 | 966 | 0.012 |
|  | GO:0005622 | C | intracellular | 90 | 4116 | 0.024 |

The up-DEFs and down-DEFs represented the significant enriched GO terms of common up- and down-DEGs in IRBB5, IRBB21 and IRBB54 respectively. The P, F and C in the ontology column were on behalf of biological process, molecular function and cellular component respectively.

Table S4. Up-DEFs and down-DEFs enriched by the DEGs with R-motif in IRBB5, IRBB21 and IRBB54

|  | GO term | Ontology | Description | Number of genes | Number in BG/Ref | FDR |
| --- | --- | --- | --- | --- | --- | --- |
| Down-DEFs in IRBB5 | GO:0006810* | P | transport | 29 | 1638 | 0.00035 |
|  | GO:0051234* | P | establishment of localization | 29 | 1638 | 0.00035 |
|  | GO:0051179* | P | localization | 29 | 1651 | 0.00035 |
|  | GO:0050794* | P | regulation of cellular process | 30 | 1955 | 0.0022 |
|  | GO:0050789* | P | regulation of biological process | 30 | 2027 | 0.0034 |
|  | GO:0065007* | P | biological regulation | 30 | 2125 | 0.0064 |
|  | GO:0007165 | P | signal transduction | 8 | 240 | 0.0085 |
|  | GO:0009987 | P | cellular process | 111 | 12839 | 0.036 |
|  | GO:0003700 | F | transcription factor activity | 13 | 651 | 0.045 |
|  | GO:0005215 | F | transporter activity | 16 | 941 | 0.045 |
|  | GO:0005623 | C | cell | 65 | 5945 | 0.0025 |
|  | GO:0044464 | C | cell part | 65 | 5945 | 0.0025 |
|  | GO:0005737 | C | cytoplasm | 21 | 1263 | 0.0052 |
|  | GO:0016020 | C | membrane | 28 | 2115 | 0.015 |
| Down-DEFs in IRBB21 | GO:0065007* | P | biological regulation | 34 | 2125 | 1.80E-05 |
|  | GO:0050789* | P | regulation of biological process | 32 | 2027 | 2.90E-05 |
|  | GO:0050794* | P | regulation of cellular process | 31 | 1955 | 2.90E-05 |
|  | GO:0007165 | P | signal transduction | 10 | 240 | 8.90E-05 |
|  | GO:0019222 | P | regulation of metabolic process | 21 | 1664 | 0.022 |
|  | GO:0044267 | P | cellular protein metabolic process | 31 | 2877 | 0.022 |
|  | GO:0010467 | P | gene expression | 27 | 2424 | 0.022 |
|  | GO:0006464 | P | protein modification process | 23 | 1929 | 0.022 |
|  | GO:0006810* | P | transport | 20 | 1638 | 0.023 |
|  | GO:0060255 | P | regulation of macromolecule metabolic process | 20 | 1640 | 0.023 |
|  | GO:0043412 | P | macromolecule modification | 23 | 1979 | 0.023 |
|  | GO:0051234* | P | establishment of localization | 20 | 1638 | 0.023 |
|  | GO:0051179* | P | localization | 20 | 1651 | 0.023 |
|  | GO:0010468 | P | regulation of gene expression | 19 | 1577 | 0.029 |
|  | GO:0006350 | P | transcription | 19 | 1629 | 0.039 |
|  | GO:0005623 | C | cell | 55 | 5945 | 0.012 |
|  | GO:0044464 | C | cell part | 55 | 5945 | 0.012 |
| Up- DEFs in IRBB21 | GO:0009056 | P | catabolic process | 9 | 433 | 0.0098 |
|  | GO:0006091 | P | generation of precursor metabolites and energy | 6 | 243 | 0.026 |
|  | GO:0005975 | P | carbohydrate metabolic process | 11 | 833 | 0.026 |
| Down-DEFs in IRBB54 | GO:0006464 | P | protein modification process | 42 | 1929 | 0.00061 |
|  | GO:0043412 | P | macromolecule modification | 42 | 1979 | 0.00061 |
|  | GO:0065007* | P | biological regulation | 42 | 2125 | 0.0021 |
|  | GO:0050789* | P | regulation of biological process | 40 | 2027 | 0.0025 |
|  | GO:0050794* | P | regulation of cellular process | 38 | 1955 | 0.0043 |
|  | GO:0044267 | P | cellular protein metabolic process | 50 | 2877 | 0.0045 |
|  | GO:0019222 | P | regulation of metabolic process | 33 | 1664 | 0.0061 |
|  | GO:0006350 | P | transcription | 32 | 1629 | 0.0077 |
|  | GO:0060255 | P | regulation of macromolecule metabolic process | 31 | 1640 | 0.016 |
|  | GO:0010468 | P | regulation of gene expression | 29 | 1577 | 0.024 |
|  | GO:0006810* | P | transport | 30 | 1638 | 0.024 |
|  | GO:0051234* | P | establishment of localization | 30 | 1638 | 0.024 |
|  | GO:0051179* | P | localization | 30 | 1651 | 0.024 |
|  | GO:0016301 | F | kinase activity | 35 | 1750 | 0.013 |
|  | GO:0044428 | C | nuclear part | 6 | 97 | 0.043 |

*indicate the common enriched GO terms among the three down-regulated DEGs list. The up-DEFs and down-DEFs were the significant enriched GO terms of up-DEGs and down-DEGs respectively. The P, F and C in the ontology column were on behalf of the GO ontology biological process, molecular function and cellular component respectively.

Table S5. The 223 curated agronomic traits-controlled genes used for the side effects analysis of gene pyramiding.

|  |  |  | Log2Fold change | | |  |
| --- | --- | --- | --- | --- | --- | --- |
| Function | Gene symbol | MSU_Locus | IRBB5 vs.IR24 | IRBB21 vs.IR24 | IRBB54 vs. IR24 | Genes with R motif |
| plant height, plant shape and tiller number | d61; OsBRI1 | LOC_Os01g52050 |  |  | -1.09138 |  |
|  | d35; OsKO2; OsKOL2; OsKOS3 | LOC_Os06g37364 | 5.19089 |  | 3.82001 |  |
|  | d18; OsGA3ox2 | LOC_Os01g08220 | -1.15146 |  |  |  |
|  | d50;5PTase | LOC_Os02g27620 |  | -1.88278 | -2.45244 | * |
|  | D53 | LOC_Os11g01330 |  |  | -1.32443 |  |
|  | sdt; OsmiR156h | LOC_Os06g44034 | -2.62083 | -2.4453 | -2.57991 |  |
|  | DGL1 | LOC_Os01g49000 | -1.03082 | -2.39966 | -1.55808 |  |
|  | Psd1 | LOC_Os01g60740 |  |  |  |  |
|  | DBS1; OsNACK | LOC_Os01g33040 |  |  |  |  |
|  | HTD2; D88; D14; qPPB3 | LOC_Os03g10620 |  |  |  | * |
|  | D3; SOLS | LOC_Os06g06050 |  |  |  | * |
|  | OsNIP3;1; DTE1 | LOC_Os10g36924 |  |  |  | * |
|  | OsCCD7; htd1; sd-t; dit1 | LOC_Os04g46470 |  |  |  |  |
|  | RIM1 | LOC_Os03g02800 |  |  |  |  |
|  | WDL1 | LOC_Os11g48070 |  |  |  |  |
|  | Brd2; lhdd10 | LOC_Os10g25780 |  |  |  |  |
|  | d6; OSH15;Oskn3 | LOC_Os07g03770 |  |  |  | * |
|  | SSD1 | LOC_Os03g19080 |  |  |  |  |
|  | sd1; OsGA20ox2; qSD1-2 | LOC_Os01g66100 |  |  |  |  |
|  | OsCYP96B4; OsDSS1; sd37; bsh1 | LOC_Os03g04680 |  |  |  |  |
|  | brd1;OsDWARF; OsBR6ox | LOC_Os03g40540 |  |  |  | * |
|  | DDF1 | LOC_Os06g04710 |  |  |  |  |
|  | GID1 | LOC_Os05g33730 |  |  |  |  |
|  | GID2 | LOC_Os02g36974 |  |  |  |  |
|  | D1; RGA1; D89 | LOC_Os05g26890 |  |  |  |  |
|  | DWT1 | LOC_Os01g47710 |  |  |  |  |
|  | D-h; HD1 | LOC_Os01g10460 |  |  |  |  |
|  | OsSIN | LOC_Os03g22510 |  |  |  |  |
|  | OsBZR1 | LOC_Os07g39220 |  |  |  |  |
|  | PAY1 | LOC_Os08g31470 |  |  |  |  |
|  | LPA1; OsIDD14 | LOC_Os03g13400 |  |  |  | * |
|  | OsMCA1; PAD | LOC_Os03g06120 |  |  |  | * |
|  | THIS1 | LOC_Os01g54810 |  |  |  |  |
| leaf color and leaf shape | AL2; CRS1 | LOC_Os09g19850 |  |  |  |  |
|  | TCD5 | LOC_Os05g34040 |  |  |  |  |
|  | YSS1 | LOC_Os04g59570 |  |  |  |  |
|  | WSL12; OsNDPK2 | LOC_Os12g36194 | -2.45542 | -2.23626 | -3.42447 |  |
|  | WSP1 | LOC_Os04g51280 |  |  | -1.23409 |  |
|  | WLP1 | LOC_Os01g54540 |  |  |  |  |
|  | ASL2; RPL21c | LOC_Os02g15900 | 1.04275 | 1.06502 |  |  |
|  | Se5; OsHY1; OsHO1; ygl2; grc1 | LOC_Os06g40080 |  | -1.19716 |  |  |
|  | YL1 | LOC_Os02g05890 | -1.31067 | -1.54862 | -1.24634 |  |
|  | LYL1 | LOC_Os02g51080 |  |  |  |  |
|  | BGL11(t) | LOC_Os11g38040 | -2.77189 | -2.68554 | -2.95883 |  |
|  | NYC1 | LOC_Os01g12710 |  |  | -1.69558 |  |
|  | NOL | LOC_Os03g45194 | -1.50762 | -1.4619 | -2.24912 |  |
|  | YLC1; OsV5A | LOC_Os09g21250 | -1.1087 | -2.32767 | -2.74288 |  |
|  | OsPORB; FGL; PGL10 | LOC_Os10g35370 |  |  |  | * |
|  | NAL9; VYL; ClpP | LOC_Os03g29810 |  |  | -1.47524 |  |
|  | OsAld-Y; ygdl-1 | LOC_Os06g40640 |  |  |  |  |
|  | YGL138(t) | LOC_Os11g05552 |  |  |  |  |
|  | YGL8 | LOC_Os01g17170 |  |  |  |  |
|  | ygl8 | LOC_Os01g73450 |  |  |  |  |
|  | YGL1; ygl80 | LOC_Os05g28200 |  |  |  |  |
|  | Chl1; OsChlD; ygl3; Ygl7; ygl98 | LOC_Os03g59640 |  |  |  |  |
|  | Chl9; OsChlI | LOC_Os03g36540 |  |  |  |  |
|  | SPL28 | LOC_Os01g50770 |  | -2.01764 | -2.6691 |  |
|  | Spl11 | LOC_Os12g38210 |  |  |  |  |
|  | OsHsfA4d; Spl7 | LOC_Os05g45410 | -1.39258 |  | -1.2356 |  |
|  | spl5; SF3b3; 0sSL5 | LOC_Os07g10390 |  |  | -1.0955 |  |
|  | SPL29; UAP1 | LOC_Os08g10600 |  |  |  |  |
|  | SPL3; OsEDR1; OsACDR1; OsMAPKKK1 | LOC_Os03g06410 | -1.55476 | -1.37346 |  |  |
|  | Fd-GOGAT1; lc7; ABC1; spl23 | LOC_Os07g46460 |  |  |  |  |
|  | SRL2; AVB; NRL2 | LOC_Os03g19520 | -1.5701 |  |  | * |
|  | SRL1 | LOC_Os07g01240 |  |  |  |  |
|  | SLL1 | LOC_Os09g23200 |  |  |  |  |
|  | CFL1 | LOC_Os02g31140 |  |  |  |  |
|  | RL14 | LOC_Os10g40960 | -1.48141 |  |  |  |
|  | ADL1; OsDEK1 | LOC_Os02g47970 |  |  |  | * |
|  | OsCSLD4; NRL1; ND1; sle1; DNL1; OsCD1 | LOC_Os12g36890 |  |  |  |  |
|  | OsAGO7 | LOC_Os03g33650 |  |  |  |  |
|  | ACL1 | LOC_Os04g33860 |  |  |  |  |
|  | ACL2 | LOC_Os02g33330 |  | 3.65825 | 1.94451 |  |
|  | Roc5; oul1 | LOC_Os06g35970 |  |  |  |  |
|  | OsZHD1; ACL-D | LOC_Os09g29130 |  |  |  | * |
|  | OsARVL4 | LOC_Os04g33580 |  |  |  |  |
|  | REL2 | LOC_Os10g41310 |  |  |  |  |
|  | REL1 | LOC_Os01g64380 |  |  |  |  |
|  | DL; OsYABBY | LOC_Os03g11600 |  |  |  |  |
|  | NAL3; OsWOX3A | LOC_Os12g01120 |  |  |  |  |
|  | NAL2; OsWOX3A | LOC_Os11g01130 |  |  |  |  |
|  | NAL1; qFLW4; LSCHL4; NAL5 | LOC_Os04g52479 |  |  |  |  |
|  | OsLG1 | LOC_Os04g56170 |  |  |  |  |
| Leaf aging and necrosis | LTN1; OsPHO2; OsUBC35 | LOC_Os05g48390 |  |  |  | * |
|  | OsNaPRT1; LTS1 | LOC_Os03g62110 |  |  | -1.93727 |  |
|  | RLS3 | LOC_Os03g38990 |  |  |  |  |
|  | RLS1 | LOC_Os02g10900 |  |  |  |  |
|  | NLS1 | LOC_Os11g14380 |  |  | -1.47394 | * |
| Leaf inclination | LC2; OsVIL3 | LOC_Os02g05840 |  |  |  | * |
|  | OsGH3.1; OsGH3-1; LC1 | LOC_Os01g57610 |  |  |  |  |
| Flowering phase | OsFKF1 | LOC_Os11g34460 |  | -1.3657 |  | * |
|  | OsCO3 | LOC_Os09g06464 |  |  | -1.29873 | * |
|  | OsHUB2; FRRP1 | LOC_Os10g41590 |  |  |  | * |
|  | HDR1 | LOC_Os02g55080 |  |  |  | * |
|  | OsCOL4 | LOC_Os02g39710 |  |  |  |  |
| Pollen development | Wda1 | LOC_Os10g33250 |  |  |  |  |
|  | RIP1 | LOC_Os12g03822 |  |  |  |  |
|  | S-27 | LOC_Os08g31219 |  |  |  |  |
|  | pss-1 | LOC_Os08g02380 |  |  |  |  |
|  | Osnop | LOC_Os06g40570 |  |  |  |  |
|  | Sa; SaM; SaF | LOC_Os01g39680 |  |  |  |  |
|  | S-28 | LOC_Os04g25540 |  |  |  |  |
|  | CAP1 | LOC_Os02g04840 |  |  |  | * |
|  | OsABCG15; PDA1 | LOC_Os06g40550 |  |  |  |  |
| Floral organ development | OsFTIP1 | LOC_Os06g41090 |  |  |  |  |
|  | AID1 | LOC_Os06g08290 |  |  |  |  |
|  | RFT1; FT-L3 | LOC_Os06g06300 |  |  |  |  |
|  | PTB1 | LOC_Os05g05280 |  |  |  |  |
|  | OsCRY1b | LOC_Os04g37920 |  |  |  | * |
|  | OSINV4 | LOC_Os04g33720 |  |  |  |  |
|  | OsCRY1a | LOC_Os02g36380 |  |  |  | * |
|  | RTS | LOC_Os01g70440 |  |  |  | * |
|  | TAW1 | LOC_Os10g33780 |  |  |  | * |
|  | OsADF | LOC_Os10g03660 |  |  |  |  |
|  | FON4; FON2 | LOC_Os11g38270 |  |  |  |  |
|  | CFO1; OsMADS32 | LOC_Os01g52680 |  |  |  |  |
|  | OsFOR1; PGIP | LOC_Os07g38130 |  |  |  |  |
|  | FON1 | LOC_Os06g50340 | 2.48489 |  |  |  |
|  | OsMADS16; SPW1 | LOC_Os06g49840 |  |  |  |  |
|  | FOS1 | LOC_Os02g21890 |  |  |  |  |
|  | DFO1; CCP1 | LOC_Os01g12890 |  |  |  |  |
|  | OsMADS1; LHS1; AFO | LOC_Os03g11614 | -2.90314 | -3.40898 | -3.08375 | * |
|  | OsJAG | LOC_Os01g03840 |  |  |  |  |
| Heading date | SDG724; lvp1; OsSET34 | LOC_Os09g13740 |  |  | -2.66602 |  |
|  | Hd17; Ef7; OsELF3; OsELF3-1; OsELF3.1; Hd-q | LOC_Os06g05060 |  |  | -1.02506 |  |
|  | Hd6; CK2α | LOC_Os03g55389 |  |  | -1.39385 | * |
|  | Hd16; CKI; EL1 | LOC_Os03g57940 |  |  | -1.28229 |  |
|  | Hd1 | LOC_Os06g16370 |  |  |  |  |
|  | qHd1 | LOC_Os01g69830 |  |  |  |  |
|  | Hd3a; FT | LOC_Os06g06320 |  |  |  |  |
|  | Ghd2 | LOC_Os02g49880 |  |  |  |  |
|  | DTH2 | LOC_Os02g49230 |  |  |  |  |
|  | DTH3;OsMADS50; OsSOC1; | LOC_Os03g03070 |  |  |  |  |
|  | DTH7; Ghd7.1; OsPRR37 | LOC_Os07g49460 |  |  |  |  |
|  | DTH8; Ghd8; OsHAP3H; LHD1; EF8 | LOC_Os08g07740 |  |  |  |  |
|  | Ehd1 | LOC_Os10g32600 |  |  |  |  |
|  | Ehd2; RID1; OsId1; Ghd10 | LOC_Os10g28330 |  |  |  |  |
|  | Ehd3 | LOC_Os08g01420 |  |  |  | * |
|  | Ehd4 | LOC_Os03g02160 |  |  |  |  |
|  | Hd18 | LOC_Os08g04780 |  |  |  |  |
|  | HAF1 | LOC_Os04g55510 |  |  |  | * |
|  | RFL; APO2 | LOC_Os04g51000 |  |  |  |  |
|  | HGW | LOC_Os06g06530 |  |  |  |  |
|  | OsFCA | LOC_Os09g03610 |  |  |  | * |
| Spike sprouting | OsCRTISO; ZEBRA2; PHS3; ZL2; MHZ5 | LOC_Os11g36440 |  |  |  |  |
|  | β-OsLCY; zebra524 | LOC_Os02g09750 |  |  | -1.2212 |  |
|  | OsPDS | LOC_Os03g08570 |  |  | -1.32846 |  |
|  | OsZDS | LOC_Os07g10490 |  |  |  |  |
| Spike shape | LP; EP3 | LOC_Os02g15950 |  |  | -1.37519 |  |
|  | LP1 | LOC_Os09g28300 |  |  |  |  |
|  | DEP1; DN1; qPE9-1; qNGR9 | LOC_Os09g26999 |  |  |  |  |
|  | DEP3; OspPLAIIIδ | LOC_Os06g46350 |  |  |  |  |
|  | SP1; OsNPF4.1 | LOC_Os11g12740 |  |  |  |  |
|  | OsLIS-L1; ASP1; OsREL2 | LOC_Os08g06480 |  |  |  | * |
|  | FZP; BFL1 | LOC_Os07g47330 |  |  |  |  |
|  | OsValRS2; WP1 | LOC_Os07g06940 |  |  |  |  |
|  | MFS1 | LOC_Os05g41760 |  |  |  |  |
|  | OsIDS1 | LOC_Os03g60430 |  |  |  | * |
|  | SUI2 | LOC_Os05g48060 |  |  |  |  |
|  | SUI3 | LOC_Os01g49024 |  |  |  |  |
|  | sui1; OsPSS-1 | LOC_Os01g02890 |  |  |  | * |
|  | EUI1; i-sd-1(t) | LOC_Os05g40384 |  |  |  | * |
| Cold tolerance at booting stage | Ctb1 | LOC_Os04g52830 |  |  |  |  |
|  | CTB4a | LOC_Os04g04330 |  |  |  | * |
| Seed shattering | SHAT1 | LOC_Os04g55560 |  |  |  |  |
|  | OsYABBY2; OsYAB2; OsSh1 | LOC_Os03g44710 |  |  |  |  |
|  | SH5 | LOC_Os05g38120 |  |  |  | * |
|  | sh4; SHA1 | LOC_Os04g57530 |  |  |  |  |
|  | qSH1 | LOC_Os01g62920 | 1.4227 |  |  | * |
| grain shape and grain weight | OsGRF4; GS2; GL2; PT2 | LOC_Os02g47280 |  |  |  | * |
|  | OsMKK4; SMG1 | LOC_Os02g54600 |  |  |  |  |
|  | DLT; OsGRAS-32;D62; GS6; SMOS2 | LOC_Os06g03710 |  |  |  | * |
|  | SRS5; TID1 | LOC_Os11g14220 | -1.20487 |  |  |  |
|  | GL3.2 | LOC_Os03g30420 |  |  |  |  |
|  | D2; CYP90D2; smg11 | LOC_Os01g10040 |  |  | 1.84531 |  |
|  | SRS3; OsKinesin-13A; sar1 | LOC_Os05g06280 |  |  |  |  |
|  | DEP2; EP2; SRS1 | LOC_Os07g42410 |  |  |  | * |
|  | GS5 | LOC_Os05g06660 | -1.90103 |  | -2.62763 |  |
|  | GIF1; OsCIN2 | LOC_Os04g33740 |  |  |  |  |
|  | PGL1 | LOC_Os03g07510 |  |  |  |  |
|  | PGL2 | LOC_Os02g51320 |  |  |  |  |
|  | d11; CYP724B1; CPB1; sg4; cl4 | LOC_Os04g39430 |  |  |  | * |
|  | BG1 | LOC_Os03g07920 |  |  |  |  |
|  | SG1 | LOC_Os09g28520 |  |  |  |  |
|  | WTG1; OsOTUB1 | LOC_Os08g42540 |  |  |  |  |
|  | qSW5; GW5 | LOC_Os05g09520 |  |  |  |  |
|  | GL7; GW7 | LOC_Os07g41200 |  |  |  |  |
|  | GW2 | LOC_Os02g14720 |  |  |  | * |
|  | GW6a; OsglHAT1 | LOC_Os06g44100 |  |  |  |  |
|  | qGW8; OsSPL16 | LOC_Os08g41940 |  |  |  |  |
|  | TGW6 | LOC_Os06g41850 |  |  |  |  |
| Grain number per panicle | LAX2; Gnp4 | LOC_Os04g32510 |  |  |  |  |
|  | Ghd7 | LOC_Os07g15770 |  |  |  |  |
|  | Gn1a; OsCKX2 | LOC_Os01g10110 |  |  | -1.3632 |  |
| Grain quality | OsSSIIIa; Flo5 | LOC_Os08g09230 |  |  |  |  |
|  | ALK; SSIIa | LOC_Os06g12450 |  |  |  |  |
|  | OsSSI; SSS1 | LOC_Os06g06560 |  |  |  |  |
|  | RSR1 | LOC_Os05g03040 |  |  |  |  |
|  | RAmy1A | LOC_Os02g52710 |  |  |  |  |
|  | du3; OsCBP20 | LOC_Os02g39890 |  |  |  |  |
|  | OsBEIIb | LOC_Os02g32660 |  |  |  |  |
|  | SSG4 | LOC_Os01g08420 |  |  |  |  |

Pleiotropic genes list only one trait they controls. Genes that have R-motif within their 5’ leader sequence, a typical motif of genes with altered translation during plant immune response, were marked with *.

Table S6. Primers used for detecting the integration and expression of *xa5* and *Xa21*.

| Target gene | Primer sequence | Application |
| --- | --- | --- |
| xa5/XhoIF | CCGGAGCTCGCCATTCAAGTTCTCG | Linked molecular marker of *xa5* |
| xa5/XhoIR | TGCTCTTGACTTGGTTCTCC |  |
| U1 | CGATCGGTATAACAGCAAAAC | Function marker of *Xa21* |
| I2 | TCTGATCATGCATGTTCTGTG |  |
| Xa21RT-F | GCAGGCTTAAAAACTTAGGCATTC | For *Xa21* qPCR |
| Xa21RT-R | TGAGAGTGTGTATGGTATCCAACCA |  |
| xa5RT-F | CTCGCCATTCAAGTTCTTGAG | For *xa5* qPCR |
| xa5RT-R | AGTCACACAGAACCAAAGGG |  |
| Ubiquitin-F | GCTCCGTGGCGGTATCAT | Internal control gene for qPCR |
| Ubiquitin-R | CGGCAGTTGACAGCCCTAG |  |
